# Prognostic score for predicting respiratory admissions among patients with chronic obstructive pulmonary disease in primary care: development and validation in population cohorts (Birmingham Lung Improvement Studies (BLISS))

**DOI:** 10.1136/bmj-2025-084521

**Published:** 2026-03-05

**Authors:** Rachel E Jordan, Spencer J Keene, Frits M E Franssen, David Fitzmaurice, Nicola J Adderley, Andrew P Dickens, James T Martin, Alice J Sitch, Alexandra Enocson, Sue Jowett, Richard D Riley, Martin R Miller, Brendan G Cooper, Alice Turner, Kate Jolly, Jon G Ayres, Robert Stockley, Sheila Greenfield, Stanley Siebert, Amanda Daley, K K Cheng, Frank de Vries, Emiel F M Wouters, Peymane Adab

**Affiliations:** 1 Department of Applied Health Sciences, College of Medicine and Health, University of Birmingham, Edgbaston, Birmingham, UK; 2 Cardiovascular Research Institute Maastricht (CARIM), Maastricht University Medical Center, Maastricht, Netherlands; 3 CIRO, Horn, Netherlands; 4 British Heart Foundation Cardiovascular Epidemiology Unit, Department of Public Health and Primary Care, University of Cambridge, Cambridge, UK; 5 Victor Phillip Dahdaleh Heart and Lung Research Institute, University of Cambridge, Cambridge, UK; 6 Utrecht Institute for Pharmaceutical Sciences, Utrecht University, Utrecht, Netherlands; 7 NIHR Birmingham Biomedical Research Centre, University Hospitals Birmingham NHS Foundation Trust and University of Birmingham, Birmingham, UK; 8 Observational and Pragmatic Research Institute, Midview City, Singapore; 9 Lung Function and Sleep, University Hospitals Birmingham, NHS Foundation Trust, Birmingham, UK; 10 NIHR Applied Research Collaboration West Midlands, West Midlands, UK; 11 Business School, University of Birmingham, Birmingham, UK; 12 Centre for Lifestyle Medicine and Behaviour, School of Sport, Exercise and Health Sciences, Loughborough University, Loughborough, UK; 13 Department of Respiratory Medicine, Maastricht University Medical Centre (MUMC), Maastricht, Netherlands; 14 Sigmund Freud Private University, Department of Medicine, Vienna, Austria

## Abstract

**Objective:**

To predict the two year risk of respiratory admission to hospital among individuals with chronic obstructive pulmonary disease (COPD), with the development and validation (internal and external) of a prognostic score.

**Design:**

Model development and validation in population cohorts.

**Setting:**

Birmingham Lung Improvement Studies (BLISS) cohort of new and existing patients with COPD in primary care (model development and internal validation); Evaluation of COPD Longitudinally to Identify Predictive Surrogate Endpoints (ECLIPSE) international cohort and UK primary care Clinical Practice Research Datalink (CPRD) Aurum database linked with Hospital Episode Statistics (external validation).

**Participants:**

1894 patients with new and existing COPD from BLISS cohort; 1749 patients with moderate to very severe COPD from ECLIPSE cohort; 27 340 patients with COPD from CPRD Aurum database linked with Hospital Episode Statistics.

**Main outcome measures:**

One or more respiratory admissions within two years of cohort entry for development, internal validation, and external validation in CPRD; severe exacerbation within two years for external validation in ECLIPSE cohort. The model was developed from 23 candidate predictors by using multivariable logistic regression with bootstrapping for internal validation and adjustment for overfitting and optimism. Discrimination and calibration were assessed at each stage. Net benefit of the score (clinical utility) was examined across a range clinically relevant risk thresholds compared with use of individual score components. Subgroup and sensitivity analyses were conducted in the CPRD. The BLISS score was directly compared with the Bertens’ score in the ECLIPSE cohort. Clinical implementation was explored with relevant stakeholders.

**Results:**

Six predictors were retained (age, COPD Assessment Test score, respiratory admissions in the previous 12 months, body mass index, diabetes, forced expiratory volume in 1 second % predicted) to form the BLISS score for estimating an individual’s two year risk of respiratory admission. The score had similar discrimination performance on internal validation (optimism adjusted C statistic 0.73 (95% confidence interval 0.70 to 0.77)) and external validation (ECLIPSE: C=0.73 (0.71 to 0.76); CPRD: C=0.71 (0.70 to 0.72)) and good calibration performance in the BLISS (slope=0.87 (95% confidence interval 0.73 to 1.02), CPRD (0.89 (0.85 to 0.93)), and ECLIPSE (0.92 (0.79 to 1.05) cohorts). Stratified analysis in the CPRD cohort showed that it was robust in different population subgroups. Net benefit analyses showed superiority of the BLISS score over individual predictors and the Bertens’ score (C=0.68 (0.65 to 0.71); calibration slope 0.68 (0.56 to 0.81)).

**Conclusions:**

The BLISS score showed good performance in estimating individual risk of respiratory admission (within two years) in cohorts containing patients from different settings and geographical locations and with different severities of COPD. Four of the included six variables are readily available in primary care records, and two are partially available but easy to collect. Impact evaluations are now needed to fully study use of the score in clinical care.

**Study registration:**

ECLIPSE ClinicalTrials.gov NCT00292552.

## Introduction

Chronic obstructive pulmonary disease (COPD) is a chronic respiratory disease and one of the most common long term conditions managed in primary care.^1^ ^2^ It is also one of the most expensive for healthcare systems, with a persistently high rate of hospital admissions due to acute exacerbations in response to respiratory infections and other environmental triggers.^3^ ^4^ ^5^ However, COPD is a heterogeneous condition with some patients disproportionately contributing to the high health service burden.^6^

Prognostic scores (or models or indices) are often used in medical practice to assess and communicate patients’ risk, guide management of individual patients, or stratify care at practice level.^7^ Many prognostic scores have been proposed for COPD,^8^ ^9^ ^10^ the best known of which are the BODE index,^11^ the DOSE index,^12^ and the ADO score.^13^ These multicomponent scores have been shown to predict prognosis better than single disease attributes such as severity of airflow obstruction or previous history of exacerbations or hospital admissions. However, most were developed and validated to predict mortality. A systematic review published in 2019 and including more than 400 studies showed that of the scores for predicting respiratory hospital admissions, one of the most burdensome and costly consequences worldwide, only one had a low risk of bias. Limitations included the development methods, lack of appropriate validation, and the impracticality of obtaining variables in primary care settings (such as the six minute walk test), where most patients with COPD are managed.^10^ Since then, several additional publications have become available; a summary of the most recent key studies predicting severe exacerbations is provided in supplementary table A.

One of the most relevant scores developed in primary care is the Bertens’ score, which was developed to predict moderate/severe exacerbations (described by steroid use or hospital admission) within two years among 240 patients with COPD aged 65 years and over in primary care.^14^ With only four variables, it is feasible to implement and had good discrimination and good calibration in the derivation cohort (C=0.75) but more moderate performance in validation cohorts—C statistic of 0.66 (95% confidence interval 0.62 to 0.71) in a primary care cohort of patients with COPD aged ≥50 years from the Netherlands^14^; 0.65 in the ICE COLD-ERIC cohort of primary care patients with COPD aged ≥40 years (Global Initiative for Chronic Obstructive Lung Disease (GOLD) stage 2-4) from Switzerland and the Netherlands.^15^ None of the available scores is routinely used in primary care, and guidelines emphasise the need for a good quality and useful prognostic score with variables that are practical to collect.^16^ ^17^ With suitable thresholds, an accurate risk score could guide clinical management at a practice level, such as frequency of reviews and referral pathways, or individually to contribute to therapeutic decisions. In the GOLD strategy for the management of COPD, a two factor matrix is recommended to guide decisions on treatment such as inhaled corticosteroids.^17^ One of these dimensions is the number of previous exacerbations or hospital admissions. The second dimension is an eight item self-reported COPD Assessment Test (CAT) or the Medical Research Council (MRC) breathlessness scale.^18^ ^19^ A more accurate risk score could be beneficial if straightforward to use clinically, but this research agenda has received limited empirical evaluation.

We present the development and internal and external validation of a new prognostic model, the BLISS (Birmingham Lung Improvement Studies) score, designed to estimate an individual’s two year risk of respiratory admission. The score derivation uses a specifically recruited primary care COPD cohort in the West Midlands, UK.^20^ Subsequent cohorts were then obtained for external validation: firstly, one of the most well known and well characterised multi-country secondary/tertiary care COPD cohorts in the world—the Evaluation of COPD Longitudinally to Identify Predictive Surrogate Endpoints COPD (ECLIPSE) cohort^21^—and then the extensive Clinical Practice Research Datalink (CPRD) Aurum database of nationally representative routinely collected UK primary care data.^22^ We additionally explored the potential of our score to be used in clinical practice by consulting with key stakeholders.

## Methods

This study is reported in accordance with the TRIPOD (Transparent Reporting of a multivariable prediction model for Individual Prognosis Or Diagnosis) statement.^23^ The overall aim of the study was to develop and validate a prognostic score that supports targeting of primary care interventions and reduces the risk of hospital admission among people with COPD. Specific objectives were to develop and internally validate a prognostic score to estimate the risk of acute respiratory hospital admissions within two years among patients with COPD by using data from the Birmingham COPD cohort; to externally validate the score in the ECLIPSE international cohort and a cohort of nationally representative patients with COPD from CPRD Aurum primary care records; to describe the net benefit (clinical utility) over individual components of the score; to compare its performance against the most relevant existing score developed and validated in primary care (the Bertens’ score)^14^; to evaluate its performance in specific subgroups of the population; and to explore clinical implementation and potential thresholds of the new prognostic score.

### Development and internal validation

Details of the Birmingham COPD cohort have been described previously.^20^ Briefly, patients aged ≥40 years with a diagnosis of COPD, listed on Quality and Outcomes Framework registers (drawn from standard diagnostic codes for COPD) of 71 UK general practices in the West Midlands, UK (prevalent COPD cases), were invited to take part in the cohort. They received an invitation with two reminders from their own general practitioner. In addition, patients aged 40-79 years without a previous diagnosis of COPD from 54 of the 71 practices were invited to take part in a case finding trial (TargetCOPD).^24^ Those responding positively to a respiratory symptom questionnaire were offered diagnostic spirometry. Any incident cases identified through this trial (either through general practice records as above or through in-trial assessments using UK recommended spirometric criteria (forced expiratory volume in 1 second (FEV_1_)/forced vital capacity (FVC) <0.7 after bronchodilator) in the presence of indicative chronic respiratory symptoms) were also invited to be part of the cohort study.^20^ The third group included in the cohort to provide a total of 2305 patients were participants of the case finding trial who reported relevant chronic respiratory symptoms but did not meet the criteria for airflow obstruction. We excluded the third group from this analysis as they did not have a diagnosis of or meet criteria for COPD. Baseline assessments (details below) for the cohort took place at cohort entry (31 May 2012 to 25 June 2014) and follow-up assessments from June 2015 to August 2016, with linked hospital episode data obtained through NHS Digital for the period 1 April 2012 to 31 March 2016.

#### Data collection in cohort study

At cohort entry, participants completed a face-to-face baseline clinical assessment and self-reported questionnaires including data on sociodemographic variables (age, sex, ethnicity, smoking status, social contact), disease specific variables (number of exacerbations in the previous 12 months (defined by courses of steroids and/or antibiotics taken for acute worsening of respiratory symptoms), presence of chronic bronchitis,^19^ extent of dyspnoea (MRC scale)^19^), and selected physician diagnosed conditions and medications. Disease specific health related quality of life was measured using the CAT^18^ and general health using a five point Likert-type scale. Self-reported exercise levels were reported using the International Physical Activity Questionnaire (IPAQ)-short and exercise capacity measured using the sit-to-stand test.^25^ ^26^ Height was measured to the nearest 0.1 cm using a Leicester height monitor and weight to the nearest 0.1 kg using the Tanita BC-420SMA body composition scale.

Lung function (FEV_1_) was measured using the nddEasy One Spirometer (ndd, Switzerland), administered by researchers trained to Association for Respiratory Technology and Physiology Foundation Spirometry Certificate standard before (maximum eight blows) and after (maximum six blows) inhalation of 400 µg salbutamol, stopping when repeatability within 100 mL (n=3) was achieved. The highest recording was then taken and FEV_1_ % predicted was estimated using the Global Lung Initiative equations.^27^ Bronchodilator responsiveness was defined as change between before and after bronchodilator FEV_1_ >12% and >200 mL or change between before and after bronchodilator FVC >12% and >200 mL. The Index of Multiple Deprivation 2010 score was calculated as a measure of deprivation derived from patients’ individual postcodes.^28^

We obtained data on current or main occupation by using a questionnaire administered by trained research assistants, who used information on skill content and skill level to assign a four digit standard occupational classification (SOC 2010)^29^ code using the CASCOT (Computer Assisted Structured Coding Tool) software. History of ever having occupational exposure to vapours, gases, dust, and fumes was derived using a job exposure matrix modified for use with SOC 2010 codes.^30^

#### Candidate predictors for BLISS score

We identified a large pool of potential candidate predictors from the literature. We selected a final set of 23 candidate predictors before analysis through discussion with a consensus panel of study investigators/clinicians, taking into consideration the available sample size and their likely contribution to the model, accuracy, and practicality in collecting the data in the primary care setting (table 1).

**Table 1:** Candidate variables for BLISS score development with data source

Description	Form of variable	Data source
**Demographics**		
Body mass index	Categorical	Cohort assessment data
Age	Continuous	Cohort self-report data: questionnaires
Sex	Binary	Cohort self-report data: questionnaires
Ethnicity	Categorical	Cohort self-report data: questionnaires
**COPD specific risk factors**		
Obstruction—FEV_1_ % predicted	Continuous	Cohort assessment data
Bronchodilator responsiveness	Binary	Cohort assessment data
Dyspnoea—MRC scale	Categorical	Cohort self-report data: questionnaires
Disease specific HRQL—CAT	Continuous	Cohort self-report data: questionnaires
Previous respiratory hospital admissions (12 months before baseline)	Binary	NHS Digital
Course of antibiotics/steroids within previous 12 months	Binary	Cohort self-report data: questionnaires
Chronic cough and/or chronic phlegm for ≥3 months of year	Binary	Cohort self-report data: questionnaires
**Other risk factors**		
Smoking	Categorical	Cohort self-report data: questionnaires
Social isolation	Binary	Cohort self-report data: questionnaires
Exercise capacity—sit-to-stand test	Continuous	Cohort assessment data
Physical activity—IPAQ	Categorical	Cohort self-report data: questionnaires
History of cardiovascular disease	Binary	Cohort self-report data: questionnaires
Medication for cardiovascular disease	Binary	Cohort self-report data: questionnaires
Heart failure	Binary	Cohort self-report: questionnaires
Asthma	Binary	Cohort self-report data: questionnaires
Depression	Binary	Cohort self-report data: questionnaires
Diabetes	Binary	Cohort self-report data: questionnaires
Any cancer	Binary	Cohort self-report data: questionnaires
Osteoporosis	Binary	Cohort self-report data: questionnaires

CAT=COPD Assessment Test; COPD=chronic obstructive pulmonary disease; FEV_1_=forced expiratory volume in 1 second; HRQL=health related quality of life; IPAQ=International Physical Activity Questionnaire; MRC=Medical Research Council.

#### Outcomes

We obtained data on hospital episodes from NHS Digital by using patients’ NHS numbers and linked them to the cohort data via a unique study ID. The primary outcome was one or more acute respiratory admissions up to two years after entry to the cohort, defined using specific respiratory ICD-10 (international classification of diseases, 10th revision) codes (see web appendix 1). We chose to use a broad measure that would capture all severe exacerbations requiring hospital admission, even if not specifically coded, and chose hospital admissions rather than all exacerbations as this was easier to define and would represent the most significant burden to the patient and the health services. A two year timeframe was compatible with the most relevant previous score and others (supplementary table A),^14^ allowed for seasonal and annual variability in infections and admissions, and would ensure sufficient time for intervention.

#### Statistical analyses

We used Stata for the analyses. The Stata code for model development and internal validation is available at https://github.com/JamesTMartin11/Bliss-Stata-Code.

We modelled the primary outcome by using multivariable logistic regression, as the outcome status (linked via NHS Digital) was known for all participants by two years. Firstly, we developed and fitted the full model, including all candidate predictor variables (table 1), and then used backward elimination, with a standard conservative significance level of 0.157 used for retention, akin to using Akaike’s information criteria.^31^ For categorical variables, we used the category with the lowest P value to assess the significance level. We modelled continuous variables on a continuous scale. Initially, we assumed a linear trend for each continuous variable, and then, where possible, we considered non-linear trends by using fractional polynomials with P<0.001 indicating the use of a fractional polynomial.^32^ We also considered fractional polynomials for the continuous variables eliminated from the model.

We used multiple imputation using chained equations for all variables considered in the model and auxiliary variables to aid imputation and combined them by using Rubin’s rules (using the MFPMI command in Stata). The number of imputed datasets used was equal to the fraction of participants with any missing data (64%).^33^ Outcomes were not imputed.

We assessed the apparent predictive performance of the fitted model by estimating calibration and discrimination in the same data used for model development. We produced a calibration plot comparing observed and estimated risk (including a smoothed calibration curve from locally estimated scatterplot smoothing) and quantified it by using the calibration slope (ideal value 1), calibration-in-the-large (the extent that predictions are systematically too low or too high; ideal value 0), and expected/observed events (ideal value 1) calculated with their confidence intervals. To judge discrimination of estimated risks between patients with and without a two year admission, we calculated the area under the receiver operating curve (the C statistic).

We examined the overfitting (optimism) in the predictive performance of the developed model by using internal validation via non-parametric bootstrapping. We used each imputed dataset to generate 100 bootstrapped datasets. We then used each one of these bootstrapped datasets to develop a prognostic model in the same way as the original model (including backward selection of predictors). We obtained estimates of apparent model performance (C statistic and calibration slope) by applying the bootstrap model fitted to the bootstrap development dataset and then estimates of test performance by applying the bootstrap model to the original dataset. The difference in apparent and test performance estimates defined the optimism for each performance statistic. For each imputed dataset, we averaged all the 100 estimates of optimism to give the mean optimism, and then averaged these across all imputed datasets. We then subtracted this average optimism from the apparent performance estimates of the original model to give optimism adjusted performance statistics.

We then used the optimised adjusted calibration slope as a uniform shrinkage factor to adjust the original model equation for overfitting. We adjusted each of the β coefficients from the original apparent model by multiplying by the shrinkage factor. These modified β coefficients were then held fixed and the intercept re-estimated to ensure calibration-in-the-large; this gave the final model (BLISS score).

As a sensitivity analysis, we developed a prognostic model with an additional four variables in the candidate pool. Owing to the documented controversies with the use of the FEV_1_ % predicted measure, we examined two alternative measures of FEV_1_ as potential predictors: FEV_1_Quotient and FEV_1_/height^2^.^34^ We also considered two other variables, Index of Multiple Deprivation score and exposure to vapours, gases, dust, and fumes, as they may be predictive of respiratory related hospital admission but may not be readily available. We also did the analyses using only the prevalent cases, and we developed a prognostic model to predict occurrence of one or more acute respiratory admissions during the full follow-up period from cohort entry until the NHS Digital admissions data was obtained (1 April 2016).

On the basis of methodology guidance available at the start of the study, we aimed to ensure that about 25 predictor parameters could be considered for model inclusion on the basis of the traditional rule of thumb of about 10 events per candidate variable.^35^ This sample size led to a uniform shrinkage factor of 0.873 (see Results), which is close to the 0.9 recommended in sample size guidance proposed more recently since our study started.^36^

### External validation of BLISS score

We obtained two further datasets after model development, to allow external validation of the BLISS score.

#### ECLIPSE validation cohort

The methodology of the ECLIPSE study has been summarised elsewhere.^21^ Briefly, 2138 patients aged 40-75 years with a previous diagnosis of moderate-to-severe COPD were recruited from December 2005 until February 2010; 1817 had the outcome recorded at the two year time point needed for this analysis. Patients with a post-bronchodilator FEV_1_ of <80% of the predicted value, baseline post-bronchodilator FEV_1_/FVC of ≤0.7, and smoking history of ≥10 pack years were recruited from 46 secondary and tertiary care centres in 12 countries in North America and Europe. We planned to use multiple imputation if more than 5% of individuals were missing one or more predictor variables. In the event, only 68/1817 (3.7%) were missing at least one predictor and therefore imputation was not needed.

We calculated the BLISS score for each individual in the ECLIPSE data. However, one of the predictors in the BLISS score was the CAT score, which was not collected in ECLIPSE; therefore, we converted the St George’s Respiratory Questionnaire for COPD (SGRQ-C)^37^ to the CAT score by using the regression equation developed by Jones and colleagues.^38^ In addition, the predictor of “respiratory admission in the previous 12 months” was not available but replaced with a similar variable: any moderate/severe exacerbation in the previous 12 months, in which moderate exacerbation was defined as patients needing prescription of antibiotics and/or systemic corticosteroids and severe exacerbation as those needing hospital admission.^39^ Depression was assessed using the Center for Epidemiologic Studies Depression Scale (CES-D),^40^ a self-administered questionnaire that measures the presence of depression in the previous week. Occupational exposure was defined as “Ever been exposed to fumes in work.” Exercise capacity was evaluated using the six minute walking distance test.^41^ Diagnosed comorbidities were collected through self-reported questionnaire.

In the ECLIPSE cohort, exacerbation assessments were made at each participant visit by using case report forms supplemented by monthly phone calls. Moderate and severe exacerbations were defined as above. The main outcome for this validation study was at least one severe exacerbation within two years. We quantified the predictive performance of the BLISS score by using the C statistic for discrimination, calibration plots, and measures, as described above.

#### CPRD validation cohort

We obtained cohort data from CPRD Aurum linked with Hospital Episode Statistics (HES) Admitted Patient Care (APC) data. CPRD Aurum comprises routinely collected electronic health records from contributing primary care practices that use EMIS Web electronic medical records software. It includes anonymised longitudinal medical records for more than 38 million patients eligible for HES linkage from 1332 primary care practices in England, with a median follow-up time for patients of 8.7 years.^42^ CPRD Aurum contains data on diagnoses, consultations, symptoms, tests/investigations, referrals, and prescriptions and is generalisable to the English population. We extracted primary care data by using the Data Extraction for Epidemiological Research tool.^43^ Linked HES APC data contain details of all admissions to NHS secondary healthcare providers in England; diagnoses are recorded using ICD-10 codes.^44^

We used a population based, retrospective, fixed cohort design to conduct external validation of the BLISS prognostic score. Patients aged 40 years or over on 1 January 2018 with a clinical diagnosis of COPD were included and followed up until 31 December 2019. We selected this time period to optimise completeness of the main variables but with an end date before the start of the covid-19 pandemic, when admission rates for COPD exacerbations were not typical. Eligibility for inclusion was restricted to patients with a minimum registration period of 12 months and a COPD diagnosis (see web appendix 2 for clinical codes) at least 12 months before study entry and a minimum follow-up period of two years or record of mortality before 31 December 2019.

Data items extracted and used in the analysis were age, sex, ethnic group, Index of Multiple Deprivation (derived from individual patients’ postcodes), smoking status, body mass index within the previous 12 months (or derived from height and weight measures), FEV_1_ % predicted (latest within previous 12 months), MRC score (latest within previous 12 months),^19^ CAT score (latest within previous 12 months),^18^ number of courses of antibiotics (amoxicillin, doxycycline, clarithromycin, co-amoxiclav, co-trimoxazole, erythromycin, levofloxacin) or steroids (prednisolone) in the previous 12 months, any acute respiratory admission in the previous 12 months (web appendix 1), and any previous record of asthma, depression, diabetes (type 1 or type 2), cancer, osteoporosis, or cardiovascular disease. We extracted all variables from the CPRD Aurum primary care data, except for respiratory admissions which we obtained from linked HES APC data.

We derived the BLISS score by using the data provided in the CPRD records for the six predictors needed. The primary outcome was one or more acute respiratory admissions up to two years after entry to the cohort, defined using the same specific respiratory ICD-10 codes as in the BLISS development cohort (web appendix 1). Outcome data came from CPRD Aurum-HES linked data. We quantified the predictive performance of the BLISS score by using the C statistic for discrimination, calibration plots, and measures.

Although the proportion of missing data in CPRD was substantial for some predictors, insufficient auxiliary information was available from other observed variables to permit reliable multiple imputation. Therefore, we did not use imputation. The performance of the model is assessed in individuals who had complete predictor information, so the current model is valid to be used only on this basis (see discussion about potential implementation).

### Comparing performance of BLISS score and Bertens’ score in ECLIPSE cohort

We compared the performance of the BLISS score with that of the Bertens’ score by using data from the ECLIPSE cohort. The Bertens’ score was developed in 240 patients with COPD aged ≥65 years selected from 51 general practices in the Netherlands from 2001 to 2003.^14^ It includes four predictors: one or more exacerbations in the previous 12 months, FEV_1_ % predicted, smoking pack years, and history of vascular disease (defined as stroke, minor stroke, or peripheral arterial disease). We extracted all data from the ECLIPSE cohort and applied the published equation to the cohort (risk of COPD exacerbation within the next 24 months=1/(1+exp−(−1.33+1.62×previous exacerbation−0.05×FEV_1_ (% predicted, per 5% interval change)+0.15×2log(pack years)+0.65×history vascular disease)). However, as peripheral arterial disease was not available in the ECLIPSE dataset, we defined vascular disease as a history of any cardiovascular disease (excluding high blood pressure) or stroke. We defined exacerbation history as symptomatic deterioration requiring pulsed oral steroid use or admission to hospital. We quantified the performance of the Bertens’ score for predicting at least one severe exacerbation within two years by using the C statistic for discrimination, calibration plots, and measures, as described above.

### Evaluating performance of BLISS score in subgroups of CPRD population and sensitivity analyses

We evaluated the performance of the BLISS score (discrimination and calibration as above) in several subgroups of the CPRD cohort: male versus female patients, four age categories, and four ethnic group categories. We also evaluated the performance of the score among patients with less than two years of follow-up and those for whom the body mass index, FEV_1_, and CAT score were recorded up to three years before baseline.

### Clinical utility of BLISS score: decision curve analysis

We used decision curve analysis in the development and two validation datasets to calculate the net benefit (clinical utility) of using the BLISS score across a range of clinically relevant risk thresholds.^45^ We also compared net benefit with strategies using individual components, classifying all patients as having high risk (treat all), or classifying all patients as having low risk (treat none). At a particular risk threshold, net benefit is the difference between the proportion of true positives and the proportion of false positives weighted by the odds of the selected threshold for high risk, where: net benefit=(true positives/N – false positives/N)×(threshold probability/1−threshold probability). In the ECLIPSE validation dataset, we also compared the BLISS score with the Bertens’ score.

### Exploring clinical implementation of BLISS score

We held two online stakeholder engagement meetings that included 10 participants identified through professional networks and their contacts—six primary care clinicians, two respiratory consultants, a primary care nurse, and a community pharmacist from a range of countries and healthcare systems—to explore their views on how the BLISS score might be used to guide patient management and whether specific thresholds could be identified to classify risk and allocate interventions. We used a broad topic guide, including the following questions. What would you use the BLISS score for? What factors need to be considered for cut points? Where would you set cut points? What intervention would you attach to each level of risk? Two of the authors (RJ and PA) alternated facilitation and note taking to identify key themes after the first group, which were then summarised for further discussion in the subsequent group. We recorded these online stakeholder groups and described key points in the results rather than presenting formalised themes. We intended these to be conversations to gather early insights rather than formal focus groups. The findings are therefore presented as indicative observations rather than formalised themes, with the intention that they may be explored further in future implementation work.

Using data from the BLISS development cohort, we then presented over a range of clinically plausible thresholds of risk (10-50%) the proportions needed to receive a particular intervention and the proportion of all hospital admissions that could potentially be affected by any intervention, at each threshold of risk.

### Patient and public involvement

A COPD patient advisory group in Birmingham was created and consulted two or three times a year at each stage of the research cycle during the development of the BLISS score (chair Michael Darby), from design of processes and patient facing materials through to dissemination of results. Patients were remunerated in line with National Institute for Health and Care Research guidelines.^46^ A clinician stakeholder group was consulted as described above.

## Results

### Characteristics of participants in development and validation cohorts

**Fig 1:**
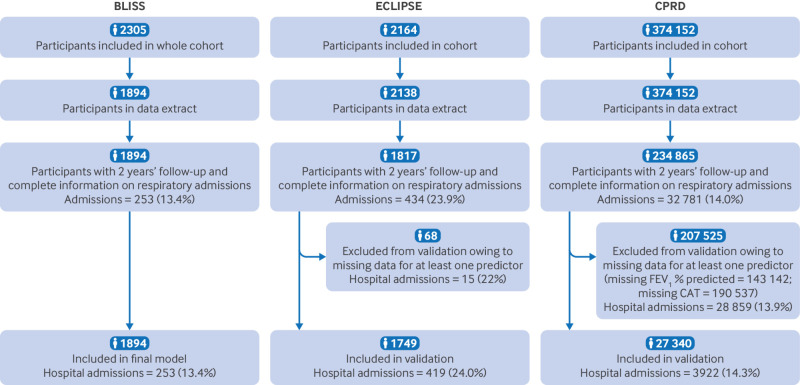
Flow of participants through each cohort. BLISS=Birmingham Lung Improvement Studies; CAT=COPD Assessment Test; COPD=chronic obstructive pulmonary disease; CPRD=Clinical Practice Research Datalink; ECIPSE=Evaluation of COPD Longitudinally to Identify Predictive Surrogate Endpoints; FEV_1_=forced expiratory volume in 1 second

Of 7176 people invited to the Birmingham COPD cohort, 1564 participants with prevalent COPD and 330 with incident COPD completed baseline assessments and were included in these analyses. Median follow-up was 3.0 (interquartile range 2.7-3.4) years, 367/1894 (19.4%) had a respiratory admission recorded during the study period, and 253/1894 (13.4%) had a respiratory admission within the primary two year period (table 2; fig 1). Although the cohort participants were more likely to be male, the age and ethnicity profile of the BLISS cohort was similar to the full CPRD COPD patient dataset (supplementary table B), which reasonably reflects the diagnosed COPD population in UK primary care.

**Table 2:** Baseline characteristics of BLISS cohort study participants for derivation of BLISS score and ECLIPSE and CPRD patients for external validation. Values are numbers (percentages) unless stated otherwise

Variable	Included BLISS population (n=1894)	Included ECLIPSE population (n=1817)*	Included CPRD population (n=27 340)
**Sociodemographic variables**			
Median (IQR) age, years	69.0 (63.2-75.2)	64 (59-69)	70 (63-76)
Male sex	1165 (61.5)	1179 (64.9)	14 855 (54.3)
Ethnicity:			
White British	1591 (84.0)	1780 (98.0)	25 301 (92.5)
Asian	36 (1.9)	4 (0.2)	424 (1.6)
African/Caribbean	14 (0.7)	27 (1.5)	195 (0.7)
Other/unclear/missing	253 (13.4)	6 (0.3)	1420 (5.2)
Level of deprivation (IMD score):			
Median (IQR)	26.3 (14.4-42.1)	NA	NA
Fifth 1	-	-	4023/27 058 (14.9)
Fifth 2	-	-	4865/27 058 (18.0)
Fifth 3	-	-	5024/27 058 (18.6)
Fifth 4	-	-	5562/27 058 (20.6)
Fifth 5	-	-	7584/27 058 (28.0)
Social isolation	116/1749 (6.6)	NA	NA
**Lifestyle variables**			
Current smoker	507/1739 (29.2)	646 (35.6)	11 868 (43.4)
VGDF exposure†	1221/1844 (66.2)	623/1815 (34.3)	NA
Physical activity (IPAQ):			
Low activity	611/1427 (42.8)	NA	NA
Moderate activity	466/1427 (32.7)	NA	NA
High activity	350/1427 (24.5)	NA	NA
Mean (SD) BMI	28.4 (5.7); n=1788	26.6 (5.6)	28.0 (6.2)
**General medical/health variables**			
Asthma	704/1648 (42.7)	421/1815 (23.2)	18 763 (68.6)
Depression	354/1609 (22.0)	441/1780 (24.8)	8185 (29.9)
Diabetes	263/1655 (15.9)	174/1815 (9.6)	5177 (18.9)
Cancer	227/1646 (13.8)	NA	3989 (14.6)
Osteoporosis	137/1546 (8.9)	233/1815 (12.8)	2457 (9.0)
Heart failure	133/1590 (8.4)	117/1815 (6.4)	NA
Heart disease	263/1626 (16.2)‡	996/1815 (54.9)§	8076 (29.5)¶
Median (IQR) exercise capacity**	18.0 (15.0-22.0) reps; n=1479	380 (305-452) m; n=1780	NA
**COPD specific factors**			
Severity of obstruction			
Median (IQR) FEV_1_ % predicted	68.8 (53.6-82.2)	48.5 (37.1-62.1)	64 (50-77)
Bronchodilator responsiveness	137 (7.2)	NA	NA
MRC dyspnoea score:			
Grade 1-2	759/1783 (42.6)	861/1764 (48.8)	15 910/27 199 (58.5)
Grade 3-5	1024/1783 (57.4)	903/1764 (51.2)	11 289/27 199 (41.5)
Median (IQR) CAT score††	19.0 (12.0-25.0); n=1400	18.6 (13.7-23.6); n=1753	13 (8-20)
Chronic cough and/or chronic phlegm	1154 (60.9)	1061 (58.4)	NA
Course of antibiotics/steroids in previous 12 m	968 (51.1)	NA	15 642/27 336 (57.2)
Respiratory hospital admission in previous 12 m‡‡			
0	1794 (94.7)	1560 (85.9)	25 665 (93.9)
1	75 (4.0)	189 (10.4)	1314 (4.8)
≥2	25 (1.3)	68 (3.7)	361 (1.3)
Any	100 (5.3)	257 (14.1)	1675 (6.1)
Respiratory hospital admission in 2 year follow-up period (primary outcome) §§	253 (13.4)	434 (23.9)	3922 (14.3)

BMI=body mass index; CAT=COPD Assessment Test; COPD=chronic obstructive pulmonary disease; CPRD=Clinical Practice Research Datalink; ECIPSE=Evaluation of COPD Longitudinally to Identify Predictive Surrogate Endpoints; FEV_1_=forced expiratory volume in 1 second; IMD=Index of Multiple Deprivation; IPAQ=International Physical Activity Questionnaire; IQR=interquartile range; MRC=Medical Research Council; NA=not available; SD=standard deviation; VGDF=vapours, gases, dusts, and fumes.

* Slightly fewer ECLIPSE participants (n=1749) were included in validation; however, baseline characteristics were similar.

† Defined as “Ever been exposed to fumes in work” for ECLIPSE.

‡ Self-reported diagnosis of coronary heart disease.

§ Self-reported diagnosis of any cardiovascular disease (including stroke, but not including high blood pressure).

¶ Diagnosed ischaemic heart disease, heart failure, stroke/transient ischaemic attack.

** Defined using sit-to-stand test in BLISS and six minute walking distance test in ECLIPSE.

†† CAT score was predicted from St George’s Respiratory Questionnaire in ECLIPSE by using a regression equation.

‡‡ Exacerbations requiring hospital admission in first year of follow-up in ECLIPSE.

§§ In BLISS and CPRD datasets, hospital admission for respiratory related problem in previous 12 months came from Hospital Episode Statistics.

Of the 2138 patients with COPD from the ECLIPSE external validation cohort, 1817 had the primary endpoint recorded (fig 1); only 67/1817 (3.7%) had one or more missing predictors, so we did not use multiple imputation. The validation analysis included 1749 patients with records of required predictors and outcomes, of whom 419 (24.0%) had at least one severe exacerbation within two years. The 68 excluded participants had a slightly lower event rate (n=15; 22%). Compared with the development cohort, the validation cohort overall were younger and slightly more likely to be male, to be current smokers, and to report higher levels of osteoporosis but lower levels of diabetes (table 2). They had worse lung function and a higher rate of previous exacerbations (although measures were not strictly comparable), but similar levels of dyspnoea, chronic cough/phlegm, and CAT scores.

Of 374 152 patients with COPD in the CPRD dataset, 234 865 had complete follow-up for two years; 207 525 individuals had one or more missing predictors (143 142/234 865 (60.9%) were missing FEV_1_ % predicted values in the previous 12 months and 190 537/234 865 (81.1%) were missing CAT scores). The validation analysis finally included 27 340 patients with records of required predictors and outcomes, of whom 3922 (14.3%) had at least one acute respiratory admission within two years (fig 1). This event rate was similar to that of the excluded patients (28 859/207 525; 13.9%). The final CPRD validation cohort was of a similar age, body mass index, diabetes prevalence, previous respiratory hospital admissions and exacerbations, and outcome event rate (two year admissions) to the BLISS cohort, but they were more likely to be female, white British, and a current smoker and to have a record of asthma or depression, milder dyspnoea, lower CAT scores, and a diagnosis of heart disease (although definitions were different from the other two cohorts) (table 2).

Compared with all patients with COPD in the CPRD dataset, the included participants were broadly similar although less likely to be female or from ethnic minorities but more likely to record a diagnosis of asthma or use of oral antibiotics/steroids for an exacerbation in the previous 12 months and slightly less likely to report an admission in the previous 12 months (supplementary table B).

In all cohorts, participants with hospital admissions were likely to be older and to report more severe airflow obstruction, worse dyspnoea, worse quality of life, and exacerbations in the previous year (supplementary tables C-E).

### Model development and internal validation in BLISS cohort

Of 23 candidate predictors, six were retained in the developed model (table 3): age, CAT score, respiratory admission in the previous 12 months, body mass index, self-report of diabetes, and FEV_1_ % predicted. Agreement was good between observed and predicted probabilities, as expected in the development dataset (see calibration plot in supplementary figure A). Internal validation via bootstrapping (accounting for the backward selection), estimated a uniform shrinkage factor of 0.873, and we applied this to obtain the final prediction model (BLISS score). The score showed promising discrimination between participants with COPD with and without a respiratory admission within two years (optimism adjusted C statistic 0.73, 95% confidence interval (CI) 0.70 to 0.77) (table 4). Individual risks of admission can be calculated using the final equation (table 3 footnote); see box 1 for an example application. Results of all sensitivity analyses can be found in the supplement.

**Table 3:** Final multivariable model (after application of uniform shrinkage factor) for risk of respiratory hospital admission within two years for participants with chronic obstructive pulmonary disease (COPD)

Variable	Odds ratio(95% CI)	β coefficients*
FEV_1_ % predicted	0.98 (0.97 to 0.98)	−0.022
Age (per year increase)	1.03 (1.01 to 1.04)	0.027
Disease specific HRQL categories (CAT score)	1.04 (1.02 to 1.06)	0.041
Previous 12 month respiratory hospital admission (presence/absence)	3.63 (2.42 to 5.45)	1.290
Diabetes (presence/absence)	1.52 (1.06 to 2.18)	0.421
Fractional polynomial transformation:		
(BMI/10)^3^	-	−0.125
(BMI/10)^3^×ln(BMI/10)	-	0.077
Constant	-	−2.491

BMI=body mass index; CAT=COPD Assessment Test; CI=confidence interval; FEV_1_=forced expiratory volume in 1 second; HRQL=health related quality of life.

* After application of uniform shrinkage factor of 0.87.

Risk score=–2.491–0.022FEV_1_%predicted+0.027age+0.041CAT+1.290previous 12 month hospital admission+0.421diabetes–0.125(BMI/10)^3^+0.077(BMI/10)^3^ln(BMI/10); ln=natural logarithm. Value −2.491 is intercept, and other numbers reflect estimated coefficients for predictors, indicating their contribution to risk. Regression coefficients represent log odds ratio for change in 1 unit in corresponding predictor. Predicted risk of hospital admission is 1/(1+e^–riskscore^).

**Table 4:** Comparing performance of BLISS score in development and external validation cohorts

Measure	BLISS cohort		ECLIPSE cohort	CPRD cohort
	Apparent performance*	Optimism corrected†		
C statistic (95% CI)‡	0.76 (0.72 to 0.79)	0.73 (0.70 to 0.77)	0.73 (0.71 to 0.76)	0.71 (0.70 to 0.72)
Calibration statistics:				
Slope (95% CI)	1.00 (0.85 to 1.15)	0.87 (0.73 to 1.02)	0.92 (0.79 to 1.05)	0.89 (0.85 to 0.93)
CITL (95% CI)	-	-	−0.15 (−0.32 to 0.02)	0.11 (0.08 to 0.15)

CI=confidence interval; CITL=calibration in the large; CPRD=Clinical Practice Research Datalink; ECIPSE=Evaluation of COPD Longitudinally to Identify Predictive Surrogate Endpoints.

* Refers to performance estimated from imputation datasets that were used to develop prediction model.

† Subtracting optimism from apparent performance.

‡ Probability that for any randomly selected pair of patients with diagnosed chronic obstructive pulmonary disease with and without respiratory hospital admission, patient with respiratory admission had higher predicted risk. Value of 0.5 represents no discrimination and 1.00 represents perfect discrimination.

Box 1 **Example of application of BLISS score (see table 3 for equation)**

A 75 year old man without diabetes, with a body mass index of 20, has had a respiratory related hospital admission in the past 12 months. The severity of his airflow obstruction (FEV_1_) was measured at 40% of predicted, and his CAT score was 30. The BLISS score estimates that he has a 66% risk of having a respiratory related hospital admission in the next two years

### External validation in ECLIPSE cohort (predicting severe exacerbations)

**Fig 2:**
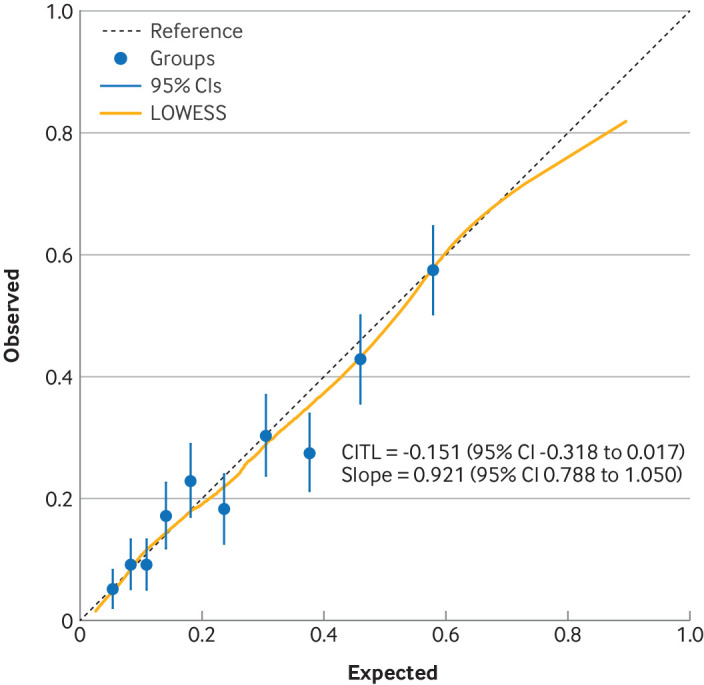
Calibration plot for BLISS score in ECLIPSE validation cohort. CI=confidence interval; CITL=calibration-in-the-large; LOWESS=locally weighted scatterplot smoothing

In the ECLIPSE external validation cohort, the discrimination of the BLISS score was similar to that in the development phase (C=0.73, 95% CI 0.71 to 0.76) (table 4). The calibration plot suggests excellent calibration across the entire range of predictions, with the calibration curve close to the line of perfect agreement, except in patients with very high predicted risks, for whom they are slightly too large. The calibration-in-the-large (−0.15 (95% CI −0.32 to 0.02) and calibration slope (0.92 (0.79 to 1.05)) (fig 2) are close to their ideal values of 0 and 1, again with slightly lower values owing to the slight overestimation at high predictions.

### External validation in CPRD cohort (predicting acute respiratory admissions)

The discrimination of the BLISS score was also similar in the CPRD cohort (C=0.71, 95% CI 0.70 to 0.72) (table 4), and the calibration slope was slightly less than 1 (0.89, 95% CI 0.85 to 0.93) (supplementary figure B).

### Comparison with Bertens’ score (predicting severe exacerbations)

Over a two year follow-up, the Bertens’ score performed less well than the BLISS score in the ECLIPSE cohort (C=0.68 (95% CI 0.65 to 0.71); calibration slope 0.68 (0.56 to 0.81)) (supplementary table F). It also performed less well over one and three year follow-up, whereas the BLISS score maintained a similar C statistic with three year follow-up and a better C statistic at one year follow-up but tended to overpredict hospital admissions at one year and underpredict at three years (supplementary figure C).

### Subgroup and sensitivity analyses

Discriminative performance in the CPRD dataset was similar between male (C=0.72, 95% CI 0.71 to 0.73) and female patients (C=0.71, 0.69 to 0.72), but we observed a trend towards performing less well in older age groups (supplementary table G). Among ethnic groups, performance seemed to be better among non-white ethnicities, although confidence intervals were wider owing to a smaller number of participants in these groups. Performance remained similar among patients with less than two years of follow-up (C=0.72, 0.71 to 0.73) and also in those with body mass index, FEV_1_, and CAT score recorded up to three years before baseline (C=0.71, 0.71 to 0.72).

Calibration slopes were slightly lower among female (0.85, 95% CI 0.79 to 0.91) than male patients (0.93, 0.87 to 0.99), suggesting a greater degree of overprediction in women. We observed a trend towards poorer calibration with increasing age, with the lowest slope seen in patients aged over 80 years (0.75, 0.65 to 0.85), indicating more marked overprediction in older adults. Among non-white ethnic groups, slopes were generally above 1, suggesting possible underprediction, although estimates were imprecise owing to small sample sizes. Coefficients are reported for sensitivity analysis of models including additional variables, using only prevalent cases and the full follow-up period in supplementary table H.

### Clinical utility of BLISS score: decision curve analysis

**Fig 3:**
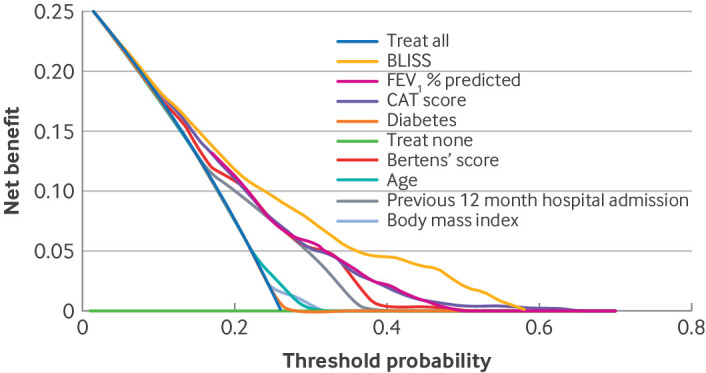
Net benefit of management using BLISS score in ECLIPSE external validation dataset to stratify risk, compared with Bertens’ score, individual components of BLISS score, treating all as high risk, or treating none. CAT=COPD Assessment Test; COPD=chronic obstructive pulmonary disease; FEV_1_=forced expiratory volume in 1 second

Decision curve analysis showed that implementing the BLISS risk score would have greater net benefit than treating all patients as being at high risk (treat all) or all as at low risk (treat none). The net benefit was also larger than for strategies using just individual components or the Bertens’ score (fig 3; supplementary figures D and E).

### Clinical implementation of BLISS score

The stakeholder group suggested several ways in which the BLISS score could be used in clinical practice. It could be used in individual consultations for communicating risk and influencing adherence to treatments. It was considered attractive for stratifying care in a population health model—for example, to guide decisions to prescribe certain treatments such as macrolides or oral steroids, to refer to other services (such as pulmonary rehabilitation, hospital admission, palliative care, integrated care, clinical pharmacists), to guide frequency of annual or post-discharge reviews, or to determine intensity of support in exacerbation action plans (self-management support, home digital monitoring, drug adherence/inhaler technique review). It could also be used to select patients for research studies or as a substitute for previous exacerbations in the GOLD Committee’s internationally recommended tool to guide risk stratified treatment.^17^

Most clinicians consulted thought that setting explicit thresholds for use might be difficult (although two strata might be most practical) because it would depend on resources/capacity available in different settings. However, it would allow the ranking of patients by risk to inform the choice of intervention or to reduce the intensity of current interventions among those at the lowest risk. Three strata could also be beneficial, focusing interventions on an intermediate risk group as those at highest risk may have social reasons necessitating admission. Finally, the BLISS score would be most useful if embedded into clinical systems and should be fully tested in practice as a next step.

Table 5 presents simplistic example scenarios for the use of different risk thresholds in the clinically relevant range to target interventions to, based on the BLISS cohort dataset. For example, if a threshold of 10% risk is chosen to designate high risk for the BLISS score, this would represent just over half of the COPD population but would represent 80% of the total respiratory admissions. A higher threshold of 15% risk would include a lower proportion of the population (approximately 30%) but represent more than two thirds of respiratory admissions.

**Table 5:** Distribution of respiratory admissions within two years by cut-point of risk of BLISS score

Risk threshold (%)	No (%) above threshold	No (%) of admissions above threshold
10	967 (51.1)	205 (81)
15	563 (29.7)	162 (64)
20	338 (17.8)	121 (48)
30	131 (6.9)	71 (28)
40	72 (3.8)	46 (18)
50	39 (2.1)	26 (10)
Total	1894 (100)	253 (100)

## Discussion

We have used data from a unique primary care COPD cohort to develop a novel prognostic score. The BLISS score has good performance relative to many other scores in predicting two year risk of respiratory admissions among patients with COPD in primary care (supplementary tables A and F). It contains six variables, of which four are readily available in primary care records and two are partially available or easy to collect. Many general practice systems have an inbuilt facility to calculate such scores, or a simple application or web page could do this. In contrast to most other published scores,^10^ the BLISS score has been developed with current best practice methods, considering a comprehensive range of potential predictors drawn from the literature and other scores, and with performance statistics at the upper range of accuracy in external datasets.^9^ The BLISS score accurately predicted the occurrence of exacerbations requiring hospital admission in the ECLIPSE cohort of patients with moderate-severe COPD with excellent calibration, indicating no need for updating in that population, and was also similarly accurate among more than 27 000 patients drawn from a large, national routine primary care database. It also performed well in different segments of the population.

### Comparison with other studies

Consistent with other evidence, age and respiratory admission in the previous 12 months were particularly important predictors of future admissions. All included predictors have previously been shown to be individually predictive and are common to many of the scores. Airflow obstruction is the most commonly found predictor, followed by previous exacerbations, age, smoking, COPD specific quality of life, body mass index, and sex.^10^

The most well known scores have been developed to predict other outcomes such as mortality or health related quality of life.^10^ Of these, the BODE index contains two of the BLISS score predictors (body mass index, obstruction) but dyspnoea and exercise capacity rather than CAT score.^11^ The DOSE index also contains two of the BLISS score predictors (obstruction and exacerbations),^12^ and the ADO score has age and obstruction in common,^13^ but both also contain dyspnoea. The CAT score and the MRC score probably measure similar dimensions (impact of breathlessness), and they are frequently used as alternatives to each other. Several scores also include comorbidities,^10^ although none identifies diabetes as a single predictive component. Diabetes is an important comorbidity that may render anyone (including people with COPD) at higher risk of hospital admission and thus could be selected in a prediction model for hospital admission in most conditions. However, specific reasons suggest that diabetes is important in COPD because its presence can influence the sputum microbiome,^47^ which in turn relates to rate of exacerbations.^48^

Very few scores have been developed in a primary care setting. However, the most relevant and well developed comparative score is that published by Bertens and colleagues,^14^ which aimed to predict exacerbations (described by steroid use or hospital admission) within two years among patients with COPD in primary care. Although it had good discrimination and good calibration in the derivation cohort (C=0.75), it had more moderate discrimination in its validation cohorts (C statistics of 0.65 and 0.66) and considered a more limited range of candidate variables than our score. We confirm similar performance of the Bertens’ score in the ECLIPSE dataset and also show that the BLISS score performed better. One advantage of the Bertens’ score, however, is that exacerbations were defined more broadly as patients needing courses of antibiotics/steroids or hospital admissions; that is, it predicts moderate exacerbations, which are more common in primary care. Our study was focused on the high risk, high cost severe exacerbations.

The more recently published ACCEPT score has many variables in common with our BLISS score,^49^ but it also, probably reflecting the more severe development trial population (at least one previous exacerbation), includes domiciliary oxygen therapy among additional variables. Furthermore, the ACCEPT score was able to include only a limited range of variables available in the three trials it was developed on and did not have sufficient information on comorbidities, for example. Performance was better in the ECLIPSE cohort than our score (C=0.77 (95% CI 0.74 to 0.80) for one or more severe exacerbations) but was not tested in a true primary care population. However, it was recently recalibrated in the ECLIPSE cohort to include a milder population of patients without previous exacerbations and tested in the TORCH trial data giving a C statistic of 0.76 (0.72 to 0.81), and a reduced version without medications or St George Respiratory Questionnaire score gave a C statistic of 0.75 (0.71 to 0.80) when predicting severe exacerbations.^50^

The PRECISE-X score was more recently developed in the CPRD database and used internal-external cross validation, obtaining a C statistic of 0.84 (95% CI 0.83 to 0.85) for five year prediction and 0.76 (0.75 to 0.77) for one year prediction of first hospital admission for acute exacerbation of COPD among only patients with newly diagnosed COPD.^51^ The final model included four mandatory predictors (sex, age, MRC dyspnoea score, and FEV_1_) and 22 optional predictors to predict time to first exacerbation in patients with newly diagnosed COPD. It has not yet been validated in other settings.

Although one of the most widely used risk scores in primary care, the QRISK3 score for cardiovascular risk, has better performance (C=0.88),^52^ our score is comparable to other scores used in primary care such as the eFI Frailty Index with a C statistic for predicting 12 month hospital admission of 0.71.^53^ It performs better than the qSOFA Score for in-hospital mortality among adults with suspected infection admitted to the intensive care unit (C=0.61).^54^

### Strengths and limitations of study

The value of including non-modifiable factors such as age in COPD prognostic scores has been debated. However, excluding such important predictive factors would lead to confounding and biased estimates of the remaining predictors, producing a score that performs badly. The aim of a prognostic score should be to predict risk accurately; the role of the clinician is to use the score to guide patient management, which can then target the modifiable factors.

Owing to controversies surrounding the best potential measure of FEV_1_,^34^ we considered two other possibilities in sensitivity analyses. However, our analyses showed that the score containing the traditional FEV_1_ % predicted gave similar performance to alternative measures evaluated in the sensitivity analyses, the measure is already commonly available, and fewer variables are needed. Another potential criticism is that the quality of spirometry in routine primary care is often considered suboptimal; however, we have shown that the BLISS score performs equally well using these measures available from routine primary care.

Unfortunately, a substantial amount of data was missing from the CPRD validation dataset, which is common in routine care data. However, although the included participants were more likely to be male and white and to have a record of asthma and courses of antibiotics or steroids to indicate exacerbations, but a slightly lower proportion of previous respiratory admissions, the remaining sociodemographic and medical factors were similar to those of the whole COPD population, and the outcome event rates were very similar among the included and excluded participants.

In routine care, the CPRD data indicate that around 40% of COPD patients have an up-to-date FEV_1_ % predicted score extractable from their records from the previous 12 months; this would be increased if data from the previous two to three years were used. Furthermore, substantial lung function information is likely to be available in text fields or attached letters that we could not access. Until recently in the UK and elsewhere, FEV_1_ was assessed approximately annually as it was used more commonly to guide treatment. Although this is no longer the case, FEV_1_ can be collected easily (and accurately with minimum coaching) using a microspirometer.^55^ Future implementation research should consider how often this is recommended to maintain an accurate risk score, although our subgroup analyses suggest that the score performs similarly even using values for FEV_1_, CAT, or body mass index from up to three years previously. The CAT score has a greater degree of missingness, but this eight point score should be easily collectable via paper, online (for example, through digital platforms such as MyCOPD (https://mymhealth.com/US)), or text based questionnaire as required for review. We could also work with commissioners to support incentivisation of the collection of these two variables. We have not evaluated the use of the score when data are missing, so the score is valid only in patients who have data available. Future work could explore how it performs in those without all the predictors. Although using either self-reported or routinely collected data has inaccuracies, we have also shown that the score performs similarly with either approach across the three cohorts, suggesting that self-reported and routinely collected data could be used interchangeably.

An advantage of the BLISS score is that it estimates risk of respiratory admissions up to two years, whereas some previous studies have follow-up limited to one year or less (supplementary table A).^9^ Unfortunately, we were unable to allow for competing risks (such as respiratory related deaths). However, only 32 (1.7%) of the development cohort participants died of any cause without having recorded an admission. We were able to include the outcomes of all patients who remained in the UK owing to the linkage with hospital admissions and mortality data; however, a small but unknown proportion may have left the UK (usually around 0.1% of this age group per year in the UK) and their events would have been under-ascertained in both the BLISS and CPRD datasets. In the CPRD dataset, we included only participants with a full two year follow-up, which would preclude those who may have died, left the practice, or lost to follow-up for other reasons.

Finally, our development population might not truly represent all primary care, as we included patients who were case-found and also those who were prepared to take part in a research study, who would be more likely to have on average milder symptoms than the average primary care population.^20^ However, despite some slight differences in the definitions of the outcome and the quality of life predictor variable, the score performed similarly well in the ECLIPSE validation cohort which was largely derived from hospital populations and importantly includes participants from 12 different countries, indicating good geographical transportability. Despite substantial missing data potentially affecting generalisability (fewer men and individuals from ethnic minorities and probably less severely affected), which might explain the tendency to over-predict in this dataset, it discriminated equivalently in CPRD Aurum-HES linked data, which as a full database is well established as being representative of the primary care population in England.^56^ We were reassured that the BLISS development cohort had a very similar ethnicity profile to the full CPRD eligible COPD population, although this does indicate that ethnic minority groups have a higher degree of missing data in primary care records, which should be remedied. We were also reassured that the event rate in the BLISS development cohort was very similar to the event rate in the CPRD dataset, which again is indicative of its representativeness. Finally, the BLISS score should also ideally be validated regularly to monitor performance over time.

### Implications for policy, practice, and research

In terms of future research direction, our rigorous approach to the development of the BLISS score and the evidence of its transportability across different COPD severities, settings, and geographical locations, suggest that now is the time to move to the next phase and evaluate its usefulness in clinical practice to guide decision making. This has been highlighted as a priority for primary care research.^57^

Impact research studies with formal economic evaluations are needed to assess the effectiveness and cost effectiveness of using the score to guide or stratify patient management. Good quality impact studies are rare; ideally, they would have a thorough co-development phase comprising both qualitative discussions with key stakeholders and quantitative analyses to identify and ascertain risk-benefit thresholds for specific pathways as exemplified in the articles by Yebyo and colleagues and Aschmann and colleagues.^15^ ^58^ Implementation strategies would be co-developed using appropriate implementation science frameworks, followed by feasibility studies integrated within the health system and, if viable, a full randomised trial with control group and economic analysis. The potential drawbacks (for example, misclassification or diverting resources away from other prevention activities with lower risk groups) should also be assessed.

Our stakeholder panel suggested several ways in which the score could be used in practice. For example, patients at greatest risk of admission might receive certain therapies, more frequent clinical reviews, and prioritised access to pulmonary rehabilitation or other services such as integrated care or intensity of self-management support. However, decisions on risk thresholds should involve formal consultations at local level with relevant stakeholder groups such as primary care staff, commissioners, managers, and patients because they will depend on local resources, services, and capacity.

Good implementation will require having sensible risk cut-off limits to enable a balance between risk and resources available to manage people at high risk, and systems in place to review patients and assign them to different management pathways. If referring to different parts of the health system, challenges will arise from data sharing and integration, which will require robust commissioning pathways.

A further use of the BLISS score might be to guide individual patient management and communicate personal risk to facilitate shared decision making and engagement. Within the 2023 international GOLD COPD strategy, the “ABE” matrix proposed to guide treatment includes one dimension related to risk of exacerbation, in which exacerbation risk is defined by number of previous exacerbations or hospital admissions.^17^ The BLISS score had a higher net benefit than exacerbation history and could be used instead as a better marker of future risk. Further formal exploration with patients and healthcare professionals is needed to consider the most relevant interventions to be guided by the BLISS score. This would then require a series of therapeutic trials in which the score is used to select patients and also to evaluate outcomes.

### Conclusions

Using robust methods and a cohort of patients with COPD representative of primary care, we have developed an accurate prognostic score for predicting respiratory admissions within a two year timeframe. The components are either readily available or easy to collect, and the score seems to perform relatively well compared with many other published scores for predicting COPD exacerbations in different segments of the population. We have shown good external validity in an international cohort comprising COPD patients from different settings and locations and with different severities, as well as in a large national primary care database in England. Given our rigorous approach to development of the BLISS score and the evidence of its transportability across different severities, settings, geographical locations, and data provenance, we would recommend evaluating its implementation in clinical decision making rather than developing further new prognostic scores. Targeting effective interventions to patients with the greatest level of risk has the potential to reduce admissions more cost effectively.

## What is already known on this topic?

A need exists to identify patients with chronic obstructive pulmonary disease most at risk of severe exacerbations and hospital admission, to target resources and provide risk stratified management
International guidelines have highlighted the need for an accurate and practical prognostic score that could be used by general practitioners

## What this paper adds

A prediction model for respiratory admissions within two years, the BLISS score, using six easily collectable predictors, was developed and internally and externally validated
The score showed good performance relative to other scores in predicting respiratory admissions within two years in three cohorts containing patients from different settings
It is the most thoroughly developed prognostic score to predict risk of hospital admission and is generalisable to patients with chronic obstructive pulmonary disease in primary care

## Data Availability

Access to anonymised patient data from CPRD Aurum is subject to a data sharing agreement containing detailed terms and conditions of use following protocol approval from the MHRA Research Data Governance Committee (CPRD study reference 23_003158). The study specific analysable dataset is therefore not publicly available but can be requested from the corresponding author subject to research data governance approvals. The BLISS data and analytical code can also be requested from the corresponding author with appropriate permissions. The STATA code for model development and internal validation is available at https://github.com/JamesTMartin11/Bliss-Stata-Code.

## Web extra

Extra material supplied by authors

Web appendix: Supplementary materials

## Notes

Contributors: For the development and internal validation part of the study, PA and DAF were co-principal investigators for the BLISS programme, which was conceived in consultation with REJ, KKC, JGA, and RAS and with BGC, AJD, SG, KJ, SJ, MRM, RDR, WSS, and AMT as co-investigators. PA led the cohort study, AE oversaw programme management, MRM and BGC oversaw spirometry training and quality control, and RDR advised on statistical aspects for the analysis. JM did the statistical analysis with supervision from AJS. APD recruited the general practices, supervised data collection, and contributed to the data analysis. RJ, APD, and PA drafted these sections of the manuscript; AS and JM drafted the statistical methods and results sections. PA had responsibility for the final content. For the validation in the ECLIPSE cohort, SJK, FV, FF, PA, and RJ conceptualised the analysis. SJK had full access to the data, did the analysis, and wrote the first draft of these sections. FF, PA, and RJ oversaw the analysis and critically revised the text. FV, AS, and EW participated in the interpretation of the data. For the external validation in the CPRD Aurum cohort, NJA, RJ, JM, PA, SK, and FF developed the study design and conceptualised the analysis. NJA extracted the cohort dataset. NJA and JM had full access to the data. JM did the analysis with contributions from NJA. RJ, NJA, and JM interpreted the data and wrote the first draft of these sections. All authors approved the final version to be published. RJ and SK contributed equally to this paper. FF and PA are the guarantors. The corresponding author attests that all listed authors meet authorship criteria and that no others meeting the criteria have been omitted.

Transparency: The lead authors (the manuscript’s guarantors) affirm that the manuscript is an honest, accurate, and transparent account of the study being reported; that no important aspects of the study have been omitted; and that any discrepancies from the study as planned (and, if relevant, registered) have been explained.

Dissemination to participants and related patient and public communities: The results of the study will be available on the University of Birmingham website at https://www.birmingham.ac.uk/research/projects/bliss and will be distributed through usual University of Birmingham media and social media channels and via clinical and patient organisations.

Provenance and peer review: Not commissioned; externally peer reviewed.
