# Supplementary material for: Prognostic score for predicting respiratory admissions among patients with chronic obstructive pulmonary disease in primary care: development and validation in population cohorts (Birmingham Lung Improvement Studies (BLISS))
Source: BMJ. 2026 Mar 5;392:e084521. doi: 10.1136/bmj-2025-084521 (PMC12961586; doi:10.1136/bmj-2025-084521)
Supplement: Supplementary file 1 — Web appendix: Supplementary materials [file jorr084521.ww1.pdf]

Development, internal and external validation of the Birmingham Lung Improvement Studies (BLISS) prognostic score: predicting respiratory hospitalisations among primary care COPD patients

SUPPLEMENTAL MATERIAL

WEB APPENDIX 1

List of ICD10 codes used to define respiratory hospital admissions

J00-06, J09-18, J20-22, J39.3, J39.8, J39.9, J40-47, J60-70, J80-86, J90-98, R05, R06.0, R06.2, R06.5, R09.2, R09.3

ICD-10 code	Description
**Acute upper respiratory infections**	
J00	Acute nasopharyngitis [common cold]
J01	Acute sinusitis
J01.0	Acute maxillary sinusitis
J01.1	Acute frontal sinusitis
J01.2	Acute ethmoidal sinusitis
J01.3	Acute sphenoidal sinusitis
J01.4	Acute pansinusitis
J01.8	Other acute sinusitis
J01.9	Acute sinusitis, unspecified
J02	Acute pharyngitis
J02.0	Streptococcal pharyngitis
J02.8	Acute pharyngitis due to other specified organisms
J02.9	Acute pharyngitis, unspecified
J03	Acute tonsillitis
J03.0	Streptococcal tonsillitis
J03.8	Acute tonsillitis due to other specified organisms
J03.9	Acute tonsillitis, unspecified
J04	Acute laryngitis and tracheitis
J04.0	Acute laryngitis
J04.1	Acute tracheitis
J04.2	Acute laryngotracheitis
J05	Acute obstructive laryngitis [croup] and epiglottitis
J05.0	Acute obstructive laryngitis [croup]
J05.1	Acute epiglottitis
J06	Acute upper respiratory infections of multiple and unspecified sites
J06.0	Acute laryngopharyngitis
J06.8	Other acute upper respiratory infections of multiple sites

J06.9	Acute upper respiratory infection, unspecified
**Influenza and pneumonia**	
J09	Influenza due to identified zoonotic or pandemic influenza virus
J10	Influenza due to identified seasonal influenza virus
J10.0	Influenza with pneumonia, seasonal influenza virus identified
J10.1	Influenza with other respiratory manifestations, seasonal influenza virus identified
J10.8	Influenza with other manifestations, seasonal influenza virus identified
J11	Influenza, virus not identified
J11.0	Influenza with pneumonia, virus not identified
J11.1	Influenza with other respiratory manifestations, virus not identified
J11.8	Influenza with other manifestations, virus not identified
J12	Viral pneumonia, not elsewhere classified
J12.0	Adenoviral pneumonia
J12.1	Respiratory syncytial virus pneumonia
J12.2	Parainfluenza virus pneumonia
J12.3	Human metapneumovirus pneumonia
J12.8	Other viral pneumonia
J12.9	Viral pneumonia, unspecified
J13	Pneumonia due to Streptococcus pneumoniae
J14	Pneumonia due to Haemophilus influenzae
J15	Bacterial pneumonia, not elsewhere classified
J15.0	Pneumonia due to Klebsiella pneumoniae
J15.1	Pneumonia due to Pseudomonas
J15.2	Pneumonia due to staphylococcus
J15.3	Pneumonia due to streptococcus, group B
J15.4	Pneumonia due to other streptococci
J15.5	Pneumonia due to Escherichia coli
J15.6	Pneumonia due to other Gram-negative bacteria
J15.7	Pneumonia due to Mycoplasma pneumoniae
J15.8	Other bacterial pneumonia
J15.9	Bacterial pneumonia, unspecified
J16	Pneumonia due to other infectious organisms, not elsewhere classified
J16.0	Chlamydial pneumonia
J16.8	Pneumonia due to other specified infectious organisms
J17	Pneumonia in diseases classified elsewhere
J17.0	Pneumonia in bacterial diseases classified elsewhere
J17.1	Pneumonia in viral diseases classified elsewhere
J17.2	Pneumonia in mycoses
J17.3	Pneumonia in parasitic diseases
J17.8	Pneumonia in other diseases classified elsewhere
J18	Pneumonia, organism unspecified
J18.0	Bronchopneumonia, unspecified
J18.1	Lobar pneumonia, unspecified
J18.2	Hypostatic pneumonia, unspecified
J18.8	Other pneumonia, organism unspecified

J18.9	Pneumonia, unspecified
**Other acute lower respiratory infections**	
J20	Acute bronchitis
J20.0	Acute bronchitis due to Mycoplasma pneumoniae
J20.1	Acute bronchitis due to Haemophilus influenzae
J20.2	Acute bronchitis due to streptococcus
J20.3	Acute bronchitis due to coxsackievirus
J20.4	Acute bronchitis due to parainfluenza virus
J20.5	Acute bronchitis due to respiratory syncytial virus
J20.6	Acute bronchitis due to rhinovirus
J20.7	Acute bronchitis due to echovirus
J20.8	Acute bronchitis due to other specified organisms
J20.9	Acute bronchitis, unspecified
J21	Acute bronchiolitis
J21.0	Acute bronchiolitis due to respiratory syncytial virus
J21.1	Acute bronchiolitis due to human metapneumovirus
J21.8	Acute bronchiolitis due to other specified organisms
J21.9	Acute bronchiolitis, unspecified
J22	Unspecified acute lower respiratory infection
**Other diseases of upper respiratory tract**	
J39.3	Upper respiratory tract hypersensitivity reaction, site unspecified
J39.8	Other specified diseases of upper respiratory tract
J39.9	Disease of upper respiratory tract, unspecified
**Chronic lower respiratory diseases**	
J40	Bronchitis, not specified as acute or chronic
J41	Simple and mucopurulent chronic bronchitis
J41.0	Simple chronic bronchitis
J41.1	Mucopurulent chronic bronchitis
J41.8	Mixed simple and mucopurulent chronic bronchitis
J42	Unspecified chronic bronchitis
J43	Emphysema
J43.0	MacLeod syndrome
J43.1	Panlobular emphysema
J43.2	Centrilobular emphysema
J43.8	Other emphysema
J43.9	Emphysema, unspecified
J44	Other chronic obstructive pulmonary disease
J44.0	Chronic obstructive pulmonary disease with acute lower respiratory infection
J44.1	Chronic obstructive pulmonary disease with acute exacerbation, unspecified
J44.8	Other specified chronic obstructive pulmonary disease
J44.9	Chronic obstructive pulmonary disease, unspecified
J45	Asthma
J45.0	Predominantly allergic asthma
J45.1	Nonallergic asthma

J45.8	Mixed asthma
J45.9	Asthma, unspecified
J46	Status asthmaticus
J47	Bronchiectasis
**Lung diseases due to external agents**	
J60	Coalworker pneumoconiosis
J61	Pneumoconiosis due to asbestos and other mineral fibres
J62	Pneumoconiosis due to dust containing silica
J62.0	Pneumoconiosis due to talc dust
J62.8	Pneumoconiosis due to other dust containing silica
J63	Pneumoconiosis due to other inorganic dusts
J63.0	Aluminosis (of lung)
J63.1	Bauxite fibrosis (of lung)
J63.2	Berylliosis
J63.3	Graphite fibrosis (of lung)
J63.4	Siderosis
J63.5	Stannosis
J63.8	Pneumoconiosis due to other specified inorganic dusts
J64	Unspecified pneumoconiosis
J65	Pneumoconiosis associated with tuberculosis
J66	Airway disease due to specific organic dust
J66.0	Byssinosis
J66.1	Flax-dresser disease
J66.2	Cannabinosis
J66.8	Airway disease due to other specific organic dusts
J67	Hypersensitivity pneumonitis due to organic dust
J67.0	Farmer lung
J67.1	Bagassosis
J67.2	Bird fancier lung
J67.3	Suberosis
J67.4	Maltworker lung
J67.5	Mushroom-worker lung
J67.6	Maple-bark-stripper lung
J67.7	Air-conditioner and humidifier lung
J67.8	Hypersensitivity pneumonitis due to other organic dusts
J67.9	Hypersensitivity pneumonitis due to unspecified organic dust
J68	Respiratory conditions due to inhalation of chemicals, gases, fumes and vapours
J68.0	Bronchitis and pneumonitis due to chemicals, gases, fumes and vapours
J68.1	Pulmonary oedema due to chemicals, gases, fumes and vapours
J68.2	Upper respiratory inflammation due to chemicals, gases, fumes and vapours, not elsewhere classified
J68.3	Other acute and subacute respiratory conditions due to chemicals, gases, fumes and vapours
J68.4	Chronic respiratory conditions due to chemicals, gases, fumes and vapours
J68.8	Other respiratory conditions due to chemicals, gases, fumes and vapours
J68.9	Unspecified respiratory condition due to chemicals, gases, fumes and vapours

J69	Pneumonitis due to solids and liquids
J69.0	Pneumonitis due to food and vomit
J69.1	Pneumonitis due to oils and essences
J69.8	Pneumonitis due to other solids and liquids
J70	Respiratory conditions due to other external agents
J70.0	Acute pulmonary manifestations due to radiation
J70.1	Chronic and other pulmonary manifestations due to radiation
J70.2	Acute drug-induced interstitial lung disorders
J70.3	Chronic drug-induced interstitial lung disorders
J70.4	Drug-induced interstitial lung disorders, unspecified
J70.8	Respiratory conditions due to other specified external agents
J70.9	Respiratory conditions due to unspecified external agent
**Other respiratory diseases principally affecting the interstitium**	
J80	Adult respiratory distress syndrome
J81	Pulmonary oedema
J82	Pulmonary eosinophilia, not elsewhere classified
J84	Other interstitial pulmonary diseases
J84.0	Alveolar and parietoalveolar conditions
J84.1	Other interstitial pulmonary diseases with fibrosis
J84.8	Other specified interstitial pulmonary diseases
J84.9	Interstitial pulmonary disease, unspecified
**Suppurative and necrotic conditions of lower respiratory tract**	
J85	Abscess of lung and mediastinum
J85.0	Gangrene and necrosis of lung
J85.1	Abscess of lung with pneumonia
J85.2	Abscess of lung without pneumonia
J85.3	Abscess of mediastinum
J86	Pyothorax
J86.0	Pyothorax with fistula
J86.9	Pyothorax without fistula
**Other diseases of pleura**	
J90	Pleural effusion, not elsewhere classified
J91	Pleural effusion in conditions classified elsewhere
J92	Pleural plaque
J92.0	Pleural plaque with presence of asbestos
J92.9	Pleural plaque without asbestos
J93	Pneumothorax
J93.0	Spontaneous tension pneumothorax
J93.1	Other spontaneous pneumothorax
J93.8	Other pneumothorax
J93.9	Pneumothorax, unspecified
J94	Other pleural conditions
J94.0	Chylous effusion
J94.1	Fibrothorax

J94.2	Haemothorax
J94.8	Other specified pleural conditions
J94.9	Pleural condition, unspecified
**Other diseases of the respiratory system**	
J95	Postprocedural respiratory disorders, not elsewhere classified
J95.0	Tracheostomy malfunction
J95.1	Acute pulmonary insufficiency following thoracic surgery
J95.2	Acute pulmonary insufficiency following nonthoracic surgery
J95.3	Chronic pulmonary insufficiency following surgery
J95.4	Mendelson syndrome
J95.5	Postprocedural subglottic stenosis
J95.8	Other postprocedural respiratory disorders
J95.9	Postprocedural respiratory disorder, unspecified
J96	Respiratory failure, not elsewhere classified
J96.0	Acute respiratory failure
J96.1	Chronic respiratory failure
J96.9	Respiratory failure, unspecified
J98	Other respiratory disorders
J98.0	Diseases of bronchus, not elsewhere classified
J98.1	Pulmonary collapse
J98.2	Interstitial emphysema
J98.3	Compensatory emphysema
J98.4	Other disorders of lung
J98.5	Diseases of mediastinum, not elsewhere classified
J98.6	Disorders of diaphragm
J98.7	Respiratory infections, not elsewhere classified
J98.8	Other specified respiratory disorders
J98.9	Respiratory disorder, unspecified
**Symptoms and signs involving the circulatory and respiratory systems**	
R05	Cough
R06.0	Dyspnoea
R06.2	Wheezing
R06.5	Mouth breathing
R09.2	Respiratory arrest
R09.3	Abnormal sputum

WEB APPENDIX 2

List of codes used to determine diagnosis of COPD within the CPRD database

SNOMED-CT Code	Description
16003001	Giant bullous emphysema
63480004	Chronic bronchitis
68328006	Centrilobular emphysema
74417001	Mucopurulent chronic bronchitis
135836000	End stage chronic obstructive airways disease
185086009	Emphysematous bronchitis
195951007	Acute exacerbation of chronic obstructive airways disease
195953005	Mixed simple and mucopurulent chronic bronchitis
195957006	Chronic bullous emphysema
195958001	Segmental bullous emphysema
195963002	Acute vesicular emphysema
266355005	Bullous emphysema with collapse
266356006	Atrophic (senile) emphysema
313296004	Mild chronic obstructive pulmonary disease
313297008	Moderate chronic obstructive pulmonary disease
13645005	Chronic obstructive pulmonary disease
84409004	Purulent chronic bronchitis
61937009	Simple chronic bronchitis
89549007	Chronic catarrhal bronchitis
45145000	MacLeod's unilateral emphysema
390891009	Chronic obstructive pulmonary disease monitoring
390941006	Chronic obstructive pulmonary disease monitoring due
394702007	Chronic obstructive pulmonary disease follow-up
395159008	Chronic obstructive pulmonary disease leaflet given
198401000000104	Chronic obstructive pulmonary disease disturbs sleep
909721000006104	[RFC] Emphysema
940131000006102	COPD monitoring administration
940161000006106	COPD monitoring invite 3
13645005	Chr. airway obstruction NOS
760601000000107	Chronic obstructive pulmonary disease 3 monthly review
1771201000006100	Chronic obstructive pulmonary disease multidisciplinary review
1824071000006104	COPD clinical pathway protocol followed
1856491000006103	Chronic obstructive pulmonary disease monitoring in primary care
1856501000006106	Chronic obstructive pulmonary disease monitoring secondary care
1856571000006100	Chronic obstructive pulmonary disease severity
1856591000006104	Chronic obstructive pulmonary disease follow-up assessment
1882421000006109	1 COPD exacerbation in past year
1882371000006102	Shared care chronic obstructive pulmonary disease monitoring
1882431000006107	3+ COPD exacerbations in past year
1882441000006102	2 COPD exacerbations in past year
1916531000006104	Access to online patient COPD education given
848431000000106	Chronic obstructive pulmonary disease care pathway

SNOMED-CT Code	Description
857791000000103	Chronic obstructive pulmonary disease rescue pack declined
866901000000103	Eosinophilic bronchitis
716358000	Telehealth chronic obstructive pulmonary disease monitoring
704123000	Step down change in chronic obstructive pulmonary disease management plan
2009501000006106	Suitable for step down change in COPD management plan
847091000000104	Acute non-infective exacerbation of chronic obstructive pulmonary disease
285381006	Acute infective exacerbation of chronic obstructive airways disease
2009451000006109	Chronic obstructive pulmonary disease post discharge review
77690003	Interstitial emphysema
84409004	Fetid chronic bronchitis
61937009	Simple chronic bronchitis NOS
74417001	Mucopurulent chronic bronchitis NOS
185086009	Obstructive chronic bronchitis NOS
63480004	Other chronic bronchitis NOS
195957006	Chronic bullous emphysema NOS
87433001	Emphysema NOS
13645005	Chronic obstructive airways disease NOS
13645005	Other specified chronic obstructive pulmonary disease
13645005	Chronic obstructive pulmonary disease NOS
717521000000104	Chronic obstructive pulmonary disease monitoring 3rd letter
196027008	Obliterative bronchiolitis due to chemical fumes
195951007	Chron obstruct pulmonary dis wth acute exacerbation, unspec
196001008	Chronic obstruct pulmonary dis with acute lower resp infectn
390891009	COPD self-management plan given
394702007	COPD follow-up
716901000000101	Chronic obstructive pulmonary disease monitor phone invite
413845009	COPD accident and emergency attendance since last visit
198901000000105	Multiple COPD emergency hospital admissions
371611000000107	COPD patient unsuitable for pulmonary rehab - enh serv admin
371611000000107	COPD patient unsuitable for pulmonary rehabilitation
375851000000108	Refer COPD structured smoking assessment - enhanc serv admin
375911000000102	COPD structured smoking assessment declined
789661000000102	Preferred place of care for next exacerbation of COPD
783631000000109	GP OOH service notified of COPD care plan
811961000000106	COPD self-management plan agreed
810951000000101	COPD self-management plan review
826111000000109	On COPD (chr obstruc pulmonary disease) supportv cre pathway
10692761000119107	Asthma-chronic obstructive pulmonary disease overlap syndrom
87433001	Other emphysema
87433001	[X]Other emphysema
87433001	Other emphysema NOS
11932351000006106	COPD GOLD group C
11932331000006104	COPD GOLD group A
11932361000006108	COPD GOLD group D
741056003	Health education - chronic obstructive pulmonary disease

SNOMED-CT Code	Description
408501008	Admit COPD emergency
892321000000109	COPD (chronic obstructive pulmonary disease) management plan declined
185086009	Chronic obstructive bronchitis
13645005	COLD - Chronic obstructive lung disease
473202005	Optimization of medication for chronic obstructive lung disease
195957006	Emphysematous bulla
185086009	COB - Chronic obstructive bronchitis
195951007	Acute exacerbation of COPD
810951000000101	COPD (chronic obstructive pulmonary disease) self-management plan review
857811000000102	COPD (chronic obstructive pulmonary disease) written self management plan declined
10692761000119107	Asthma-COPD overlap syndrome (ACOS)
4981000	Vesicular emphysema
185086009	Obstructive chronic bronchitis
185086009	Chronic bronchitis with emphysema
196026004	Chronic emphysema caused by chemical fumes
718241000000107	Issue of COPD (chronic obstructive pulmonary disease) rescue pack
195951007	Acute exacerbation of chronic obstructive pulmonary disease
77690003	Interstitial emphysema of lung
897311000000101	Shared care COPD (chronic obstructive pulmonary disease) monitoring
4981000	Panacinar emphysema
383611000000102	At risk of COPD (chronic obstructive pulmonary disease) exacerbation
408501008	Admit COPD emergency
13645005	COPD - Chronic obstructive pulmonary disease
45145000	Unilateral emphysema
857791000000103	COPD (chronic obstructive pulmonary disease) rescue pack declined
135836000	End stage chronic obstructive pulmonary disease
408501008	Emergency hospital admission for chronic obstructive pulmonary disease
87433001	Emphysema of lung
1110861000000102	QOF (Quality and Outcomes Framework) chronic obstructive pulmonary disease quality indicator-related care invitation
77690003	Interstitial emphysema
611541000000106	Chr. airway obstruction NOS
611541000000106	Chronic obst. pulm. dis. NOS
611541000000106	Chronic obstructive pulmonary disease NOS
611541000000106	Chronic obstructive airways disease NOS
857661000000104	Seen in chronic obstructive pulmonary disease clinic
1880061000006106	COPD management plan declined
1763751000006101	GP out of hours notified of COPD care plan
270473001	History of chronic obstructive pulmonary disease
718241000000107	Issue of chronic obstructive pulmonary disease rescue pack
13645005	Chronic obst. pulm. dis. NOS
940141000006107	COPD monitoring invite 1
293991000000106	Very severe chronic obstructive pulmonary disease
4981000	Panlobular emphysema

SNOMED-CT Code	Description
185086009	Obstructive chronic bronchitis
13645005	Other specified chronic obstructive airways disease
717021000000106	Chronic obstructive pulmonary disease monitoring 2nd letter
723245007	Number of COPD exacerbations in past year
375851000000108	Referred for COPD structured smoking assessment
826111000000109	On chronic obstructive pulmonary disease supprtv cre pathway
11932341000006109	COPD GOLD group B
4981000	Alveolar emphysema of lung
473202005	Optimisation of medication for chronic obstructive lung disease
790301000000101	Referral to COPD (chronic obstructive pulmonary disease) community nursing team
195957006	Bullous emphysema
270473001	History of chronic obstructive airway disease
13645005	Chronic obstructive lung disease
408501008	Emergency hospital admission for COPD (chronic obstructive pulmonary disease)
857661000000104	Seen in COPD (chronic obstructive pulmonary disease) clinic
68328006	Centriacinar emphysema
760601000000107	COPD (chronic obstructive pulmonary disease) 3 monthly review
473202005	Optimization of medication for chronic obstructive pulmonary disease
848431000000106	COPD (chronic obstructive pulmonary disease) care pathway
10692761000119107	ACOS - asthma-chronic obstructive pulmonary disease overlap syndrome
2009511000006109	Not suitable for step down change in COPD management plan
1839331000006101	Seen in chronic obstructive pulmonary disease clinic
1656601000006103	COPD patient unsuitable for pulmonary rehabilitation
940151000006109	COPD monitoring invite 2
909711000006107	[RFC] Chronic obstructive pulmonary disease (COPD)
939991000006108	COPD medication review
851261000006100	Chronic bronchitis, acute exac
401184000	Chronic obstructive pulmonary disease monitoring by nurse
401185004	Chronic obstructive pulmonary disease monitoring by doctor
394703002	Chronic obstructive pulmonary disease annual review
313299006	Severe chronic obstructive pulmonary disease
195949008	Chronic asthmatic bronchitis
195949008	Chronic wheezy bronchitis
195959009	Zonal bullous emphysema
716241000000106	Chronic obstructive pulmonary disease monitoring 1st letter
716281000000103	Chronic obstructive pulmonary disease monitoring verb invite
414087000	Emergency COPD admission since last appointment
375911000000102	COPD structured smoking assessment declined - enh serv admin
760621000000103	COPD (chronic obstructive pulmonary disease) 6 monthly review
198401000000104	COPD (chronic obstructive pulmonary disease) disturbs sleep
371611000000107	COPD (Chronic obstructive pulmonary disease) patient unsuitable for pulmonary rehabilitation
63480004	Other chronic bronchitis
63480004	Chronic bronchitis NOS
13645005	[X]Other specified chronic obstructive pulmonary disease

SNOMED-CT Code	Description
827571000000106	Has chronic obstructive pulmonary disease care plan
897311000000101	Shared care chronic obstructive pulmonary disease monitoring
198411000000102	COPD (chronic obstructive pulmonary disease) does not disturb sleep
783631000000109	GP (general practitioner) OOH (out of hours) service notified of COPD (chronic obstructive pulmonary disease) care plan
1823851000006103	Chronic obstructive pulmonary disease confirmed
760621000000103	Chronic obstructive pulmonary disease 6 monthly review
204991000000107	Suspected chronic obstructive pulmonary disease
847091000000104	Acute non-infective exacerbation of COPD (chronic obstructive pulmonary disease)
196026004	Chronic emphysema due to chemical fumes
52571006	Chronic tracheobronchitis
13645005	Chronic obstructive airways disease
87433001	Emphysema
736283006	Chronic obstructive pulmonary disease clini management plan
713731000000102	Chronic obstructive pulmonary disease monitoring admin
790301000000101	Referral to COPD community nursing team
84409004	Fetid chronic bronchitis
77690003	Interstitial pulmonary emphysema

Supplementary Table A: characteristics of selected risk scores predicting exacerbations among COPD patients

First Author and Year	Model name	Patient setting	Cohort details	Outcome	Events/N	Selection method	Model variables	Validation	Validation result
Bertens, Loes C M 2013<br><br>Yebyo, Henock G. 2021	NA	D: Primary care<br>V: Primary care<br>V^{ICE}: Primary care	D: Primary care patients ≥aged 65 years from 51 general practices in Netherlands (April 2001 - June 2003)<br><br>V: Utrecht GP Network database of primary care patients aged ≥50 and over with 2010 entry.<br><br>V: ICE COLD ERIC: multinational study in primary care patients in Switzerland and the Netherlands, moderate to severe COPD (GOLD stages II–IV).	COPD exacerbation within 2 years	D: 70/243<br>V: 222/792<br>V^{ICE}: unclear	Backward-selection procedures, using a P-value<0.20 from the log likelihood ratio test. Only started with six preselected potential predictors.	Previous exacerbations, FEV1% predicted, pack years of smoking, history of vascular disease	External	AUC: 0.66 (95% CI: 0.62–0.71) Calibration: Plot shows good agreement<br><br>AUC^{ICE}: 0.65<br>Calibration^{ICE}: Calibration plot shows some underprediction at the lower predictions
Abu Hussein, Nebal S. 2023	NA	Primary care	Swiss COPD cohort; Questionnaire-based observational cohort study of COPD patients in general practice from 2014-2022. Included only smokers or ex-smokers with at least 20 pack years	Annual exacerbation rate	98/256	Akaike's information criterion + stepwise backward algorithm of variables available (demographic data, physical examination information, spirometric parameters, symptoms (sputum production, dyspnea), comorbidities, medical treatment history, and exacerbation history. Age,	FEV1% predicted, mMRC, LABA+ICS, LABA+LAMA+ICS, Exacerbation history	Internal only via 75%/25% splitting	AUC = 0.75 Calibration = (0.34 predicted exacerbations vs 0.28 observed exacerbations)

First Author and Year	Model name	Patient setting	Cohort details	Outcome	Events/N	Selection method	Model variables	Validation	Validation result
						gender, height, weight, body mass index, and current smoking status			
Adibi, Amin 2020	ACCEP T	D: Tertiary (mainly)<br>V: Secondary care and some tertiary	D: 3 RCTs of participants without previous or existing history of asthma, who had at least one exacerbation over the past 12 months.<br><br>V: ECLIPSE, a non-interventional, multicentre observational study	Rates of exacerbations and severe exacerbations over 1 year	D: 3056/2380(total exacerbations)<br><br>V: 996/1819(with ≥1 exacerbation)	clinical relevance and availability of predictors. No data driven selection performed.	age, sex, body-mass index, smoking status, domiciliary oxygen therapy, lung function, symptom burden (St. Georges Respiratory Questionnaire), current medication use (LABA, LAMA, ICS), statins, and exacerbation history of an individual as a prior in Bayesian approach.	External	AUC = 0·73 (95% CI 0·70–0·76) for two or more exacerbations and 0·74 (95% CI 0·70–0·78) for at least one severe exacerbation.<br><br>Calibration:<br>Overall exacerbation rates:<br>observed 1·20 events per year vs predicted 1·31 events per year.<br><br>Severe exacerbation rates: observed 0·27 events per year vs predicted 0·25 events per year
Safari, Abdollah 2022	ACCEP T 2.0	Secondary care and some tertiary	ECLIPSE used for recalibration and Towards a Revolution in COPD Health (TORCH) for external validation	Primary: ≥2 moderate or ≥1 severe exacerbation in next 12 months; Secondary: occurrence of any moderate/severe exacerbation or	Recalibration: 1646/1803 moderate; 471 severe exacerbations<br>V: 886/1091 moderate and 188 severe	See ACCEPT model above	Same as ACCEPT (above) with reduced versions created removing symptom score and/or baseline medications as predictors.	External	AUC = 0.76 (0.72-0.79);<br>Calibration: calibration-in-the-large: 0.13, mROC p-value test for miscalibration=0.38

First Author and Year	Model name	Patient setting	Cohort details	Outcome	Events/N	Selection method	Model variables	Validation	Validation result
				any severe exacerbation	exacerbations				
Chaudhary, Muhammad 2023	NA	D: Tertiary care<br>V: Tertiary care	D: SPIROMICS cohort, a multicentre study done at 12 clinical sites across the USA, of individuals aged 40–80 years enrolled and distributed across four strata according to smoking status and spirometry between Nov 12, 2010 and July 31, 2015.<br>V: the Genetic Epidemiology of COPD (COPDGene) cohort enrolled between Jan 10, 2008, and April 15, 2011	≥1 severe exacerbations within 3 years (and 2-years as secondary outcome). Other outcomes include ≥2 and ≥3 severe exacerbations in 3 years	D: 331/1956<br>V: 468/6965	a priori selection	age, sex, race, BMI, FEV1, exacerbation history, smoking status, St George's Respiratory Questionnaire, square root of airway wall area of a hypothetical airway with an internal perimeter of 10 mm and CT density gradient.	External, but also internal using bootstrapping (1000 repeats) and random splitting into disjoint into training (70%) and held-out testing (30%) at each iteration.	AUC: 0·768 (0·767–0·769)<br>Calibration: Brier score = 0.088 but calibration plots show overprediction
Chen, Xueying 2020	NA	Tertiary care	SPIROMICS (described above)	≥1 severe exacerbations at 3- and 5-years	523/1711	Smallest Akaike Information Criterion (unclear if forward or backward selection).<br><br>Candidate predictors included factors related to severe exacerbations in published literature and considered clinical ready accessibility. These included age, BMI, severe exacerbations history, comorbidities, post-	BMI, severe exacerbations in the prior year, comorbidity index, post- bronchodilator FEV1% predicted, and white blood cell counts	Internal via bootstrapping	AUC: 0.74 (95%CI: 0.71–0.76)<br>unclear if for 3- or 5-year time horizon<br>Calibration: Plots for 3- and 5-year time horizons are displayed and show good agreement. However, estimates for 3-year time horizon are too high and higher than 5-year time horizon.

First Author and Year	Model name	Patient setting	Cohort details	Outcome	Events/N	Selection method	Model variables	Validation	Validation result
						bronchodilator FEV1% predicted, mMRC and CAT			
Horne, Benjamin D. 2021	Summit Lab score	V: Secondary / tertiary care	V: retrospective analysis of a randomized, placebo-controlled, double-blind study in 61 participating centres in Germany, Norway, the Philippines, the Republic of Korea, Thailand, and the USA were to evaluate treatment effects of once-daily fluticasone furoate/vilanterol 100/25 µg in patients with COPD and an elevated arterial stiffness over 24 weeks.	moderate-to-severe exacerbation of COPD at 24 weeks	V: 40/430	both backward and forward stepwise selection (in original development study: Horne et al. Int J Chron Obstruct Pulmon Dis 2020).	baseline body mass index, number of pack years of smoking history, number of prior COPD hospitalizations, baseline FEV1, baseline heart rate, baseline systolic blood pressure, history of prior MI, prior HF diagnosis, prior diabetes diagnosis, age at baseline, and baseline prescription of antithrombotics, antiarrhythmics, and xanthines	External	No discrimination or calibration performed but the likelihood of moderate-to-severe for tertile 3 was significantly different from tertile 1 (HR 2.19; 95% CI: 1.07, 4.49; P = 0.033) but was even more significantly different for tertile 3 compared to tertile 2 (P = 0.003)

First Author and Year	Model name	Patient setting	Cohort details	Outcome	Events/N	Selection method	Model variables	Validation	Validation result
Kerkhof, Marjan 2015	NA	Primary care	Patients ≥40 years old with COPD identified from the Optimum Patient Care Research Database (OPCRD) a quality-controlled, longitudinal, respiratory-focused database containing anonymous data from general practices in the UK	≥2 exacerbations in the outcome year	D: 3,973/16,565 (in the first outcome year) V: Unclear/2,713	backward selection of the model having the lowest AIC.<br><br>Candidate predictors identified from a search of the literature and from expert opinion of the authors. These included sex, age, height, weight, BMI, smoking status, mMRC dyspnea score, CAT score, number of exacerbations in the previous year, asthma, eczema, allergic or nonallergic rhinitis, nasal polyps, diabetes mellitus, gastroesophageal reflux disease (GERD), ischemic heart disease, heart fail- ure, anxiety/depression, Charlson comorbidity index, lung function, and Peripheral blood eosinophilia	exacerbations in the baseline year, FEV1 % predicted, age, height, eosinophilia in noncurrent smokers, asthma, nonallergic rhinitis, nasal polyps, ischemic heart disease, anxiety or depression, GERD.	Internal via splitting by eligibility before (development) and after (validation) March 2013.	AUC = 0.74 (95% CI: 0.71–0.76) Calibration = Plot for validation sample showed underprediction.
Lapi, Francesco 2024	CEX-Hscore	Primary care	≥45 years old and diagnosed with COPD and data Health Search Database (HSD), an Italian general practice data source, comprising data from computer-based patient records registered by a selected group of general practitioners.	Time to first moderate and severe COPD exacerbation within 6 months	D: ~16833/42084 V: ~8780/21679	current literature, previous work of the author, and/or clinical basis.	age, smoking, diagnosis of Osteoarthritis, gastroesophageal reflux disease, and asthma, polypharmacy, prior moderate COPD exacerbations, prescription of SABA, LAMA, ICS, and	Internal by data splitting bootstrapping up to 200 random samples using the entire cohort	AUC = 0.66 (95% CI: 0.65-0.67) in validation cohort and 0.611 (0.606–0.616) after bootstrap. Calibration: Slope = 1.03 and p-value = 0.51 for hypothesis to reject "perfect" calibration in the

First Author and Year	Model name	Patient setting	Cohort details	Outcome	Events/N	Selection method	Model variables	Validation	Validation result
							fixed combinations of LABA/ICS.		validation cohort. Slope = 1.002 (0.970–1.029) after bootstrapping.
Make, Barry J. 2015 Herer, Bertrand 2018	SCOPE X	Tertiary care (mainly)	D: three double-blind, randomized, parallel-group, clinical studies of 6–12 months' duration in 3,141 patients with moderate-to-very-severe COPD aged .40 years,10 pack-years smoking, pre-bronchodilator FEV1<50% predicted, FEV1 /FVC ratio <0.70, at least one exacerbation in the previous year, and no prior asthma.<br><br>V: Consecutive inpatient or outpatient COPD patients attending a pulmonary rehabilitation program at Bligny Hospital Center, France.	time to first severe COPD exacerbation requiring treatment with oral corticosteroids and/or emergency department (ER) visit/hospitalization within 6 months	D: Unclear/3141<br>V: 32/125	Backward selection of these predictors was performed, ranked by p-value.<br><br>Unclear how candidate predictors were selected.	number of COPD maintenance medications, reliever use, number of inhalations, number of exacerbations in the previous year, sex, SGRQ, Pre-bronchodilator FEV1/FVC ratio	External	Discrimination: AUC = 0.74 (0.65-0.81)<br>Calibration: not performed.

First Author and Year	Model name	Patient setting	Cohort details	Outcome	Events/N	Selection method	Model variables	Validation	Validation result
Montserrat-Capdevila, Josep 2015	NA	Primary care	≥40 years old patients with a COPD diagnosis in 2010 from seven primary care centres of the Lleida Health Region (catchment population: 172,950) and having a spirometry test in the last two years	moderate-to-severe exacerbation of COPD in 3-years	2080/2501	Automatic forward (P<0.02) and backward (P<0.1) selection.<br><br>Candidate predictors included: age, gender, spirometry results (FEV1/CVF, FVC, FEV1), comorbidities (heart failure, ischemic heart disease, diabetes, chronic kidney failure, atrial fibrillation and anaemia), history of smoking, 23- valent pneumococcal and influenza immunisations for the 2009/10 season, years since COPD was diagnosed, number of visits to the health centre and number of acute exacerbations in the year prior to the start of the study, and COPD severity according to the GOLD Guidelines	age, gender, previous exacerbations, influenza and pneumococcal immunisations, number of previous visits to the GP and severity (GOLD)	Internal via 70%/30% splitting	Discrimination: AUC = 0.69 (95% CI: 0.64-0.74)<br>Calibration: Not reported

First Author and Year	Model name	Patient setting	Cohort details	Outcome	Events/N	Selection method	Model variables	Validation	Validation result
Sadatsafavi, Mohsen 2025	PRECISE-X	Primary care	CPRD Aurum database from 2004-2022 containing 1,491 general practice data from across the UK. Included patients were newly diagnosed with COPD, aged ≥40 years old and were either current or former smokers.	Severe exacerbations within 5 years of COPD diagnosis. Secondary outcome at 1-year	23205/219015	Hybrid approach that combined data-driven selection with expert input on predictor availability. Demographics, symptom score, lung function, socioeconomic status, eosinophils counts and other biomarkers, and comorbidities were considered.	age, sex, FEV1, and MRC + over 20 optional predictors. Only the first four predictors are needed to produce an estimate but using all is recommended, and is used for the internal-external validation	Internal-external cross-validation approach (split sample repeated with each of 9 regions removed sequentially) (Results combined using random effects meta-analysis	Discrimination: AUC = 0.82 (95% CI: 0.80-0.83) Calibration: mean calibration error (difference between average observed and predicted risk) = 2.5% (-0.8-5.7%); calibration slope = 0.997 (0.95-1.04).
Stanford, Richard H. 2018	NA	Insurance claims data	D: MarketScan database, a sample of the commercially insured U.S. population with COPD who were ≥40 years and who had at least one COPD medication dispensed during the year following COPD diagnosis, and were excluded if they had a severe COPD exacerbation in baseline period. V: The Reliant database, of adult patients receiving integrated primary and specialty care within a Massachusetts-based provider network, spanning commercial and	severe COPD exacerbation during the at-risk period (Year 2)	D: 7345/111904 V: Unclear	1000 bootstrap resampling in combination with backward stepwise variable selection. Candidate predictors included: gender, age, region, insurance plan, H1N1 flu season (October 2009 to May 2010), pulmonologist visit, county characteristics (altitude; number of pulmonologists per 100,000 inhabitants; number of hospitals per 100,000 inhabitants; proportion of households below the low-income margin; median household income; proportion of patients without health insurance; proportions of high school dropouts and college	age, use of chronic oxygen therapy, use of 4 or more canisters of rescue medication, having at least 2 prior moderate exacerbations, and type of COPD diagnoses received during the baseline period.	External	AUC = 0.71 Calibration: not reported.

First Author and Year	Model name	Patient setting	Cohort details	Outcome	Events/N	Selection method	Model variables	Validation	Validation result
			Medicare insurance.			graduates; urban/suburban/rural localization; and proportion of white, black, Hispanic, and Asian patients, derived from the Area Resource File), type of COPD diagnosis, exacerbation history (moderate only [ie, defined as outpatient treated or ED visit for COPD with a dispensing for an oral corticosteroid within 7 days]), COPD medications (based on at least 1 dispensing), concomitant medications, procedures (flu and pneumococcal vaccines, use of chronic oxygen therapy, nebulizer, and spirometry; all based on Current Procedural Terminology and Healthcare Common Procedure Coding System codes), and comorbidities based on ICD-9-CM codes.			
Valera-Novella, Elisa 2023	ESEx index	Secondary care	D: outpatients with COPD who attended regular medical visits at the Meseguer Hospital, Murcia, Spain during 2014.	at least 3 recurrent severe COPD exacerbations within 3-years	D: 20/122	Preselection of candidate predictors based on prior literature followed by forward (p ⩽ 0.156) and backward (p⩽<0.05) selection (forward selection model adjusted for history of exacerbations).<br><br>13 candidate predictors that	6-minute walking distance test, five-repetition sit-to-stand test, Number of severe exacerbations in the previous year	Internal via 500 bootstrap samples	Discrimination: AUC = 0.913 (95% CI: 0.86–0.97) Calibration: The CITL =−0.06 (95% CI: −0.89 to 0.77) and Calibration slope = 1.01 (95% CI: 0.57–1.44) Confidence intervals for calibration are

First Author and Year	Model name	Patient setting	Cohort details	Outcome	Events/N	Selection method	Model variables	Validation	Validation result
						had been associated, in previous studies, with future acute exacerbations of COPD. We only included predic-tors available in outpatient clinical settings. These included sociodemographic, clinical, pulmonary, and functional measurements.			wide.
Wang, Ye 2024	SERCO	Secondary and Tertiary care	D and V: The ACURE study, a multistage, stratified, and cluster sampling method to recruit hospitals from mainland China, including participants aged ≥18 years with confirmed or suspected hospitalisation for AECOPD attending 176 secondary and tertiary sites distributed across 29 provinces in China, selected by November 2021.	severe exacerbations and COPD- specific readmissions at 1-month, 6- month and 12- month.	D: ~254/2196<br>V: ~230/1869	backward elimination with clinical judgment. Filtering based on p-value and effect size.<br><br>Candidate predictors based on existing knowledge and literature review, the prespecified potential predictors were considered if they were clinically relevant and available. Included demographic characteristics, COPD- related disease history, comorbidities and complications, treatment during hospitalisation and clinical characteristics.	BMI, education, smoking status, prior COPD diagnosis, hospitalised exacerbations in past 12 months, prior LABA, GOLD stage, SABA during prior hospitalisation, Methylxanthine treatment during prior hospitalisation, expectorant treatment during prior hospitalisation, chronic cor pulmonale, diabetes*SABA, chronic cor pulmonale*expectorant treatment, respiratory failure*oxygen threrapy.	External from different regions compared to Development	Discrimination: 1-month: AUC = 0.65 (0.57-72); 6-months: AUC = 0.64 (0.59-0.68) 12-months: 0.64 (0.60-0.69) Calibration: plots show that SERCO underestimates the proba- bilities in lower- risk groups while overestimating in the higher- risk groups

REFERENCES

Abu Hussein, N. S., Giezendanner, S., Urwyler, P., Bridevaux, P. O., Chhajed, P. N., Geiser, T., Joos Zellweger, L., Kohler, M., Miedinger, D., Pasha, Z., Thurnheer, R., von Garnier, C., & Leuppi, J. D. (2023). Risk Factors for Recurrent Exacerbations in the General-Practitioner-Based Swiss Chronic Obstructive Pulmonary Disease (COPD) Cohort. *Journal of Clinical Medicine*, *12*(20). https://doi.org/10.3390/jcm12206695

Adibi, A., Sin, D. D., Safari, A., Johnson, K. M., Aaron, S. D., FitzGerald, J. M., & Sadatsafavi, M. (2020). The Acute COPD Exacerbation Prediction Tool (ACCEPT): a modelling study. *The Lancet Respiratory Medicine*, *8*(10), 1013–1021. https://doi.org/10.1016/S2213-2600(19)30397-2

Bertens, L. C. M., Reitsma, J. B., Moons, K. G. M., van Mourik, Y., Lammers, J. W. J., Broekhuizen, B. D. L., Hoes, A. W., Rutten, F. H., Mourik, Y. Van, Lammers, J. W. J., Broekhuizen, B. D. L., Bertens, L. C. M., Reitsma, J. B., Moons, K. G. M., van Mourik, Y., Lammers, J. W. J., Broekhuizen, B. D. L., Hoes, A. W., & Rutten, F. H. (2013). Development and validation of a model to predict the risk of exacerbations in chronic obstructive pulmonary disease. *International Journal of Chronic Obstructive Pulmonary Disease*, *8*, 493–499. https://doi.org/10.2147/COPD.S49609

Chaudhary, M. F. A., Hoffman, E. A., Guo, J., Comellas, A. P., Newell, J. D., Nagpal, P., Fortis, S., Christensen, G. E., Gerard, S. E., Pan, Y., Wang, D., Abtin, F., Barjaktarevic, I. Z., Barr, R. G., Bhatt, S. P., Bodduluri, S., Cooper, C. B., Gravens-Mueller, L., Han, M. L. K., … Reinhardt, J. M. (2023). Predicting severe chronic obstructive pulmonary disease exacerbations using quantitative CT: a retrospective model development and external validation study. *The Lancet Digital Health*, *5*(2), e83–e92. https://doi.org/10.1016/S2589-7500(22)00232-1

Chen, X., Wang, Q., Hu, Y., Zhang, L., Xiong, W., Xu, Y., Yu, J., & Wang, Y. (2020). A nomogram for predicting severe exacerbations in stable COPD patients. *International Journal of COPD*, *15*, 379–388. https://doi.org/10.2147/COPD.S234241

Herer, B., & Chinet, T. (2018). Acute exacerbation of COPD during pulmonary rehabilitation: Outcomes and risk prediction. *International Journal of COPD*, *13*, 1767–1774. https://doi.org/10.2147/COPD.S163472

Horne, B. D., Ali, R., Midwinter, D., Scott-Wilson, C., Crim, C., Miller, B. E., & Rubin, D. B. (2021). Validation of the summit lab score in predicting exacerbations of chronic obstructive pulmonary disease among individuals with high arterial stiffness. *International Journal of COPD*, *16*, 41–51. https://doi.org/10.2147/COPD.S279645

Horne, B. D., Hegewald, M. J., Crim, C., Rea, S., Bair, T. L., & Blagev, D. P. (2020). The summit score stratifies mortality and morbidity in chronic obstructive pulmonary disease. *International Journal of COPD*, *15*, 1741–1750. https://doi.org/10.2147/COPD.S254437

Kerkhof, M., Freeman, D., Jones, R., Chisholm, A., & Price, D. B. (2015). Predicting frequent COPD exacerbations using primary care data. *International Journal of Chronic Obstructive Pulmonary Disease*, *10*, 2439–2450. https://doi.org/10.2147/COPD.S94259

Lapi, F., Marconi, E., Lombardo, F. P., Cricelli, I., Ansaldo, E., Gorini, M., Micheletto, C., Di Marco, F., & Cricelli, C. (2024). Development and validation of a prediction score to assess the risk of incurring in COPD-related exacerbations: a population-based study in primary care. *Respiratory Medicine*, *227*(November 2023), 107634. https://doi.org/10.1016/j.rmed.2024.107634

Make, B. J., Eriksson, G., Calverley, P. M., Jenkins, C. R., Postma, D. S., Peterson, S., Östlund, O., & Anzueto, A. (2015). A score to predict short-term risk of COPD exacerbations (SCOPEX). *International Journal of COPD*, *10*, 201–209. https://doi.org/10.2147/COPD.S69589

Mapel, D. W., Roberts, M. H., Sama, S., Bobbili, P. J., Cheng, W. Y., Duh, M. S., Nguyen, C., Thompson-Leduc, P., Van Dyke, M. K., Rothnie, K. J., Sundaresan, D., Certa, J. M., Whiting, T. S., Brown, J. L., & Roblin, D. W. (2021). Development and validation of a healthcare utilization-based algorithm to identify acute exacerbations of chronic obstructive pulmonary disease. *International Journal of COPD*, *16*, 1687–1698. https://doi.org/10.2147/COPD.S302241

Miravitlles, M. (n.d.). Affiliations : Pr ep rin t n ot er r ed Pr ep rin t n ed. *Preprint*.

Montserrat-Capdevila, J., Godoy, P., Marsal, J. R., Barbe, F., & Galvan, L. (2015). Risk of exacerbation in chronic obstructive pulmonary disease: a primary care retrospective cohort study. *BMC Family Practice*, *16*, 173. https://doi.org/10.1186/s12875-015-0387-6

Sadatsafavi, M., Miravitlles, M., Quint, J., Perugini, V., Tavakoli, H., Amegadzie, J. E., Alcazar Navarrete, B., & Group, R. E. G. (REG)-C. W. (2025). Developmentand Validation of PRECISE-X, a Risk Prediction Model for the First Severe Exacerbation in Patients with Chronic Obstructive Pulmonary Disease. *Preprint*. https://www.ssrn.com/abstract=5167969

Safari, A., Adibi, A., Sin, D. D., Lee, T. Y., Ho, J. K., & Sadatsafavi, M. (2022). ACCEPT 2·0: Recalibrating and externally validating the Acute COPD exacerbation prediction tool (ACCEPT). *EClinicalMedicine*, *51*, 101574. https://doi.org/10.1016/j.eclinm.2022.101574

Stanford, R. H., Nag, A., Mapel, D. W., Lee, T. A., Rosiello, R., Schatz, M., Vekeman, F., Gauthier-Loiselle, M., Philip Merrigan, J. F., & Duh, M. S. (2018). Claims-Based Risk Model for First Severe COPD Exacerbation. *American Journal of Managed Care*, *24*(2), e45–e53.

Valera-Novella, E., Bernabeu-Mora, R., Montilla-Herrador, J., Escolar-Reina, P., García-Vidal, J. A., & Medina-Mirapeix, F. (2023). Development of the ESEx index: a tool for predicting risk of recurrent severe COPD exacerbations. *Therapeutic Advances in Chronic Disease*, *14*, 1–11. https://doi.org/10.1177/20406223231155115

Wang, Y., He, R., Ren, X., Huang, K., Lei, J., Niu, H., Li, W., Dong, F., Li, B., Yang, T., & Wang, C. (2024). Developing and validating prediction models for severe exacerbations and readmissions in patients hospitalised for COPD exacerbation (SERCO) in China: a prospective observational study. *BMJ Open Respiratory Research*, *11*(1), 1–12. https://doi.org/10.1136/bmjresp-2023-001881

Yebyo, H. G., Braun, J., Menges, D., ter Riet, G., Sadatsafavi, M., & Puhan, M. A. (2021). Personalising add-on treatment with inhaled corticosteroids in patients with chronic obstructive pulmonary disease: a benefit–harm modelling study. *The Lancet Digital Health*, *3*(10), e644–e653. https://doi.org/10.1016/S2589-7500(21)00130-8

Supplementary Table B: Baseline characteristics of CPRD COPD patients comparing final validation dataset with all eligible COPD patients

**Variable**	**All CPRD patients (N = 374,152)**	**All CPRD patients with 2 years follow-up (N = 234,865)**	**Finally included CPRD Patients (N = 27,340)**
**Sociodemographic Variables**			
Age, mean (SD)	69.2 (11.8)	68.9 (11.1)	69.9 (9.9)
Missing	*0*	*0*	*0*
Male Sex	191,529 (51.2)	118637 (50.5)	14855 (54.3)
Missing	*0*	*0*	*0*
Ethnicity			
White British	321978 (86.1)	205515 (87.5)	25301 (92.5)
Asian	10100 (2.7)	6308 (2.7)	424 (1.6)
African/Caribbean	4842 (1.3)	3013 (1.3)	195 (0.7)
Other/Unclear/Missing	37232 (9.9)	20029 (8.5)	1420 (5.2)
Level of deprivation (IMD Quintile)			
1	52,587 (14.3)	33285 (14.4)	4023 (14.9)
2	64,994 (17.7)	40549 (17.6)	4865 (18.0)
3	68,903 (18.8)	42772 (18.5)	5024 (18.6)
4	81,525 (22.2)	50977 (22.1)	5562 (20.6)
5	99,494 (27.1)	63175 (27.4)	7584 (28.0)
Missing	*6,649 [1.7]*	*4,107 [1.7]*	*282 [1.0]*
**Lifestyle Variables**			
Current Smoker	162,649 (43.6)	99404 (42.4)	11868 (43.4)
Missing	*788 [0.2]*	*296 [0.1]*	*0*
BMI, median (IQR)	27.3 (23.6, 31.6)	27.52 (23.9, 31.8)	27.3 (23.8, 31.495)
Missing	*127,255 [34.0]*	*84,634 [36.0]*	*0*

**General Medical/health variables**			
Asthma	194,874 (52.1)	146319 (62.3)	18763 (68.6)
Depression	117,584 (31.4)	76131 (32.4)	8185 (29.9)
Diabetes	59,434 (15.9)	41668 (17.7)	5177 (18.9)
Cancer	54,637 (14.6)	33507 (14.3)	3989 (14.6)
Osteoporosis	36,721 (9.8)	23042 (9.8)	2457 (9.0)
Cardiovascular disease	107,271 (29.2)	67652 (28.8)	8076 (29.5)
**COPD specific factors**			
Severity of obstruction (FEV1 % predicted), median [IQR]	65 (50, 77)	64 (51, 77)	64 (50, 77)
Missing	*220,201 [58.9]*	*143,142 [60.9]*	*0*
MRC Score (within one year)			
1	39028 (18.3)	27072 (19.0)	4820 (17.7%)
2	82277 (38.6)	57264 (40.1)	11090 (40.8)
3	57166 (26.9)	38256 (26.8)	7471 (27.5)
4	28409 (13.3)	17287 (12.1)	3299 (12.1)
5	6019 (2.8)	2974 (2.1)	519 (1.9)
Missing	*161,253 [43.1]*	*92012 [39.]*	*141 [0.5]*
CAT Score (within one year), median (IQR)	14 (8, 21)	13 (8, 20)	13 (8, 20)
Missing	*309,894 [82.8]*	*190,537 [81.1]*	*0*
Antibiotics/Steroids (Within last year)	169,035 (50.4)	122,964 (52.4)	15,642 (57.2)
Missing	*38,567 [10.3]*	*22 [0.0]*	*4 [0.0]*
Previous 12m Respiratory Admission	31,228 (8.3)	15,895 (6.8)	1,675 (6.1)

Missing	*0*	*0*	*0*

Values are number (percentage) unless specified. Percentages in square brackets indicate missing data for each characteristic; these are reported separately from subgroup distribution percentages and therefore do not sum to 100%.

Supplementary Table C: Baseline characteristics of BLISS cohort study participants by respiratory hospitalisation during study period.

Variable	Total population (N =1,894)	No hospitalisation (N = 1,641)	Hospitalisation (N = 253)	P-value[1]
**Sociodemographic variables**				
Age, median [IQR]	69.0 [63.2, 75.2]	68.5 [63.1, 74.7]	71.5 [64.3, 78.0]	<0.001
Male sex	1165 (61.5)	995 (60.6)	170 (67.2)	0.046
Missing	*0*	*0*	*0*	
Ethnicity				
White British	1591 (84.0)	1377 (83.9)	214 (84.6)	0.820
Asian	36 (1.9)	31 (1.9)	5 (2.0)	
African/Caribbean	14 (0.7)	11 (0.7)	3 (1.2)	
Mixed	7 (0.4)	7 (0.4)	0 (0.0)	
Other	96 (5.1)	85 (5.2)	11 (4.3)	
Unclear/Missing	150 (7.9)	130 (7.9)	20 (7.9)	
Level of deprivation (IMD score), median [IQR]	26.3 [14.4, 42.1]	25.6 [14.1, 41.6]	30.0 [16.8, 44.9]	0.019
Missing	*20*	*18*	*2*	
Social isolation	116 (6.6)	98 (6.5)	18 (7.6)	0.530
Missing	145	*130*	*15*	
**Lifestyle variables**				
Smoking				
Never Smoker	193 (11.1)	177 (11.7)	16 (7.0)	0.110
Current Smoker	507 (29.2)	437 (28.9)	70 (30.8)	
Ex Smoker	1039 (59.7)	898 (59.4)	141 (62.1)	
Missing	*155*	*129*	*26*	
VGDF exposure	1221 (66.2)	1043 (65.2)	178 (72.7)	0.022
Missing	*50*	*42*	*8*	
Physical activity (IPAQ)				
Low Activity	611 (42.8)	512 (41.0)	99 (55.9)	<0.001
Moderate Activity	466 (32.7)	416 (33.3)	50 (28.2)	
High Activity	350 (24.5)	322 (25.8)	28 (15.8)	
Missing	*467*	*391*	*76*	
BMI, mean (SD)	28.4 (5.7)	28.5 (5.5)	27.9 (6.6)	0.17
Missing	*106*	*88*	*18*	
**General Medical/health variables**				
Asthma	704 (42.7)	606 (42.1)	98 (47.1)	0.17
Missing	*246*	*201*	*45*	
Depression	354 (22.0)	304 (21.7)	50 (23.9)	0.47
Missing	*285*	*241*	*44*	
Diabetes	263 (15.9)	214 (14.8)	49 (23.2)	0.002
Missing	*239*	*197*	*42*	
Cancer	227 (13.8)	198 (13.8)	29 (13.6)	0.91
Missing	*248*	*209*	*39*	
Osteoporosis	137 (8.8)	114 (8.4)	23 (11.5)	0.15

Missing	*342*	*289*	*53*	
Coronary heart disease	263 (16.2)	219 (15.5)	44 (20.6)	0.061
Missing	*268*	*229*	*39*	
Heart failure	133 (8.4)	105 (7.6)	28 (13.4)	0.005
Missing	*304*	*260*	*44*	
Medication for CVD	982 (51.8)	838 (51.1)	144 (56.9)	0.083
Missing	*0*	*0*	*0*	
Exercise capacity (Sit to stand test), median [IQR]	18.0 [15.0, 22.0]	19.0 [15.0, 23.0]	16.0 [12.0, 20.0]	<0.001
Missing	*415*	*336*	*79*	
General health (Likert scale)				
1	121 (6.8)	111 (7.1)	10 (4.3)	<0.001
2	647 (36.2)	600 (38.6)	47 (20.3)	
3	802 (44.9)	683 (43.9)	119 (51.3)	
4	190 (10.6)	143 (9.2)	47 (20.3)	
5	28 (1.6)	19 (1.2)	9 (3.9)	
Missing	*106*	*85*	*21*	
**COPD specific factors**				
Severity of obstruction (FEV_1 % predicted), median [IQR]	68.8 [53.6, 82.2]	70.6 [55.9, 83.8]	53.3 [37.7, 69.4]	<0.001
Missing	*103*	*81*	*22*	
Bronchodilator responsiveness	137 (7.2)	109 (6.6)	28 (11.1)	0.011
Missing	*0*	*0*	*0*	
MRC Dyspnoea score				
Grade 1 – 2	759 (42.6)	702 (45.2)	57 (24.7)	<0.001
Grade 3 – 5	1024 (57.4)	850 (54.8)	174 (75.3)	
Missing	*111*	*89*	*22*	
CAT score, median [IQR]	19.0 [12.0, 25.0]	18.0 [12.0, 24.0]	24.0 [18.0, 31.0]	<0.001
Missing	*494*	*429*	*65*	
Chronic cough and/or chronic phlegm	1154 (60.9)	987 (60.1)	167 (66.0)	0.075
Missing	*0*	*0*	*0*	
Self-reported course of antibiotics/steroids in the prior 12m	968 (51.1)	816 (49.7)	152 (60.1)	0.002
Missing	*0*	*0*	*0*	
Respiratory hospitalisation in the previous 12m[2]				
0	1794 (94.7)	1589 (96.8)	205 (81.0)	<0.001
1	75 (4.0)	43 (2.6)	32 (12.6)	
2+	25 (1.3)	9 (0.5)	16 (6.3)	
Any	100 (5.3)	52 (3.2)	48 (19.0)	<0.001
Missing	*0*	*0*	*0*	

Values are Number (percentage) unless specified.

[1]: P-value obtained from t-test, Mann-Whitney U test, or chi-squared test. [2]: Hospitalisation for respiratory related problem in previous 12 months obtained from Hospital episode statistics. IQR: Inter-quartile range.

VGDF: Vapours, Gases, Dusts and Fumes

IMD: Index of Multiple Deprivation

CVD: cardiovascular disease

Supplementary Table D: Baseline characteristics of ECLIPSE COPD patients by exacerbation status within 2 years of observation time

**Characteristic**	**no exacerbations (N = 555)**	**⩾1 moderate exacerbations (N = 828)**	**⩾1 severe exacerbations (N = 434)**
Age – mean (SD)	62.9 (7.5)	63.1 (6.9)	64.0 (6.8)
Missing	0	0	0
No. males	408 (74)	489 (59)	282 (65)
Missing	0	0	0
Below High school education	202 (37)	266 (33)	185 (44)
Missing	11	22	14
Current smoker	216 (39)	284 (34)	146 (34)
Missing	0	0	0
Heart failure	42 (8)	35 (4)	40 (9)
Missing	0	2	0
Diabetes	67 (12)	73 (9)	34 (8)
Missing	0	2	0
Cardiovascular disease	323 (58)	438 (53)	235 (54)
Missing	0	2	0
Depression*	91 (17)	210 (26)	140 (33)
Missing	11	14	12
BMI (kg/m^2) – mean (SD)	27.1 (5.7)	26.6 (5.5)	25.8 (5.5)
Missing	0	0	0
≥1 exacerbation in Previous 12 months	125 (23)	434 (52)	287 (66)
Missing	0	0	0
FEV1% predicted – mean (SD)	54.6 (15.3)	49.6 (14.9)	41.5 (14.4)
Missing	3	0	1
mMRC score			
0	105 (20)	101 (13)	28 (7)
1	228 (42)	308 (38)	91 (22)
2	139 (26)	258 (32)	164 (39)
3	57 (11)	113 (14)	83 (20)

4	10 (2)	27 (3)	52 (12)
Missing	16	21	16
SGRQ-C – mean (SD)	40.6 (18.4)	46.6 (17.0)	56.2 (16.6)
Missing	17	33	14
CAT* - mean (SD)	16.2 (6.6)	18.3 (6.1)	21.8 (6.0)
Missing	17	33	14
BODE score – mean (SD)	2.3 (1.9)	2.9 (1.9)	4.2 (2.1)
Missing	22	40	31

Characteristics are expressed as n (%) unless otherwise specified. Patients in the moderate exacerbation group have not had a severe exacerbation but those in the severe exacerbation group may have had at least one moderate exacerbation. * assessed using CES-D (38), a self-administered questionnaire that measures the presence of depression in the previous week. BMI, body mass index; mMRC, modified Medical Research Council; SGRQ-C, Saint George's Respiratory Questionnaire for COPD; CAT, COPD Assessment Test; FEV, Forced Expiratory Volume in the first second; BODE (BMI, Obstruction, Dyspnoea, and Exercise Capacity).

Supplementary Table E: Baseline characteristics of CPRD COPD patients by exacerbation status within 2 years of observation time

	No Hospitalisation (N = 23,418)	Hospitalisation (N = 3922)	p-value
**Sociodemographic variables**			
Country			
England	23386 (99.9)	3922 (100.0)	0.021
Northern Ireland	32 (0.1)	0 (0.0)	
Level of deprivation (IMD Quintile)			
1	3478 (15.0)	545 (13.9)	0.39
2	4171 (18.0)	694 (17.7)	
3	4285 (18.5)	739 (18.9)	
4	4747 (20.5)	815 (20.8)	
5	6459 (27.9)	1125 (28.7)	
Age, mean (SD)	69.6 (9.9)	72.1 (9.6)	<0.001
Male Sex	12809 (54.7)	2046 (52.2)	0.003
Ethnicity			
White	21727 (92.8)	3574 (91.1)	0.002
Asian	345 (1.5)	79 (2.0)	
Black	160 (0.7)	35 (0.9)	
Other/Missing	1186 (5.1)	234 (6.0)	
**Lifestyle variables**			
Smoking			
Never Smoked	800 (3.4)	110 (2.8)	0.13
Ex-Smoker	12450 (53.2)	2112 (53.9)	
Current Smoker	10168 (43.4)	1700 (43.3)	
BMI (within one year), median (IQR)	27.4 (23.9, 31.5)	26.7 (22.9, 31.2)	<0.001
**General Medical/health variables**			
Asthma	15900 (67.9)	2863 (73.0)	<0.001
Depression	6971 (29.8)	1214 (31.0)	0.13
Diabetes	4280 (18.3)	897 (22.9)	<0.001
Cancer	3309 (14.1)	680 (17.3)	<0.001
Osteoporosis	1966 (8.4)	491 (12.5)	<0.001
Cardiovascular disease	6606 (28.2)	1470 (37.5)	<0.001
**COPD specific factors**			
FEV1 % Predicted (within one year), median (IQR)	66 (52, 78)	54 (40, 69)	<0.001
MRC Score (within one year)			
1	4486 (19.3)	334 (8.6)	<0.001
2	9860 (42.3)	1230 (31.5)	

3	6154 (26.4)	1317 (33.8)	
4	2469 (10.6)	830 (21.3)	
5	330 (1.4)	189 (4.8)	
CAT Score (within one year), median (IQR)	12 (7, 19)	17 (10, 24)	<0.001
Antibiotics/Steroids (Within last year)	12848 (54.9)	2794 (71.3)	<0.001
Previous 12m Respiratory Admission	962 (4.1)	713 (18.2)	<0.001

Characteristics are expressed as n (%) unless otherwise specified.

Supplementary Table F: Accuracy of BLISS and Bertens' model for predicting severe exacerbations in ECLIPSE cohort COPD patients.

	**BLISS score**	**Bertens' model**
**1-year exacerbation**		
N	1894	1959
AUC(95% CI)	0.76 (0.73 to 0.79)	0.70 (0.67 to 0.73)
Calibration slope (95% CI)	1.08 (0.91 to 1.2)	0.78 (0.63 to 0.93)
CITL (95% CI)	(-)0.75 (-0.92 to -0.58)	(-)1.65 (-1.78 to -1.52)
**2-year exacerbation**		
N	1749	1811
AUC (95% CI)	0.73 (0.71 to 0.76)	0.68 (0.65 to 0.71)
Calibration slope (95% CI)	0.92 (0.79 to 1.05)	0.68 (0.56 to 0.81)
CITL (95% CI)	(-)0.15 (-0.32 to 0.02)	(-)1.00 (-1.12 to -0.89)
**3-year exacerbation**		
N	547	571
AUC (95% CI)	0.73 (0.69 to 0.78)	0.69 (0.64 to 0.73)
Calibration slope (95% CI)	0.93 (0.71 to 1.14)	0.72 (0.52 to 0.93)
CITL (95% CI)	0.57 (0.28 to 0.87)	(-)0.31 (-0.49 to -0.13)

AUC: Area under the curve, akin to the c statistic

CITL: calibration in the large

Supplementary Table G: BLISS score performance in predicting hospital admissions at two-years: sub-group and sensitivity analyses within the CPRD cohort

	N	C-Statistic	95% CI	Calibration Slope	95% CI
Entire included CPRD Cohort	27,340	0.71	0.70 to 0.72	0.89	0.85, 0.93
Sex					
Females	12,485	0.71	0.69 to 0.72	0.85	0.79 to 0.91
Males	14,855	0.72	0.71 to 0.73	0.93	0.87 to 0.99
Age					
<60	4,529	0.72	0.70 to 0.75	0.91	0.80 to 1.02
61 to 70	8,286	0.73	0.71 to 0.75	0.98	0.90 to 1.06
71 to 80	10,222	0.70	0.68 to 0.71	0.84	0.77 to 0.90
Over 80	4,303	0.65	0.63 to 0.67	0.75	0.65 to 0.85
Ethnicity					
White	25,301	0.71	0.70 to 0.72	0.88	0.83 to 0.92
Asian	424	0.79	0.74 to 0.84	1.23	0.90 to 1.55
Black	195	0.78	0.70 to 0.85	1.16	0.70 to 1.63
Other/Missing	1420	0.72	0.69 to 0.76	0.93	0.76 to 1.10
**Sensitivity analyses**					
FEV1, BMI, & CAT score recorded in the past 3 years	36,291	0.71	0.71 to 0.72	0.90	0.86 to 0.93
Including those with < 2 years follow up	40353	0.72	0.71 to 0.73	0.90	0.87 to 0.93

Supplementary Table H: Comparison of BLISS prediction model and models from sensitivity analyses without internal validation

Predictors	BLISS Model			Additional Variables			Prevalent Cases Only			Full Follow Up Period		
	Coefficient	95% Confidence Interval		Coefficient	95% Confidence Interval		Coefficient	95% Confidence Interval		Coefficient	95% Confidence Interval	
FEV_1 %Predicted	-0.026	-0.034	-0.018				-0.023	-0.032	-0.015	-0.028	-0.035	-0.021
Age (years)	0.031	0.015	0.048	0.016	-0.001	0.033	0.037	0.019	0.055			
Age (years)/10										-6.484	-10.376	-2.591
(Age/10)*ln(Age/10)										2.371	1.036	3.705
CAT score	0.048	0.028	0.067	0.050	0.031	0.068	0.049	0.029	0.070	0.048	0.030	0.065
Previous 12 month respiratory hospitalisation	1.478	1.015	1.942	1.504	1.040	1.969	1.458	0.985	1.931	1.540	1.067	2.014
Diabetes	0.483	0.071	0.894	0.470	0.064	0.876	0.555	0.134	0.975	0.692	0.332	1.051
$(BMI/10)^3$	-0.143	-0.215	-0.071	-0.148	-0.221	-0.076	-0.139	-0.217	-0.061			
$((BMI/10)^3)*ln(BMI/10)$	0.088	0.044	0.133	0.092	0.047	0.137	0.086	0.038	0.134			
$(BMI/10)^2$										-0.803	-1.161	-0.446
$((BMI/10)^2)*ln(BMI/10)$										0.465	0.254	0.676
Heart Failure										0.359	-0.078	0.795
Current Smoker							0.695	-0.019	1.409	0.835	0.248	1.422
Ex Smoker							0.636	-0.022	1.293	0.699	0.158	1.241
FEV1Q/100				0.616	-0.007	1.240						
(FEV1Q/100)*ln(FEV1Q/100)				-1.246	-1.990	-0.501						
$FEV1/h^2$				-2.353	-3.165	-1.540						
Gender - Male				0.444	0.131	0.757						
Constant	-2.630	-4.092	-1.167	-2.153	-3.738	-0.569	-3.822	-5.573	-2.070	13.759	4.636	22.882
**C statistic (95%CI)**	**0.76 (0.72,0.79)**			**0.76 (0.73, 0.80)**			**0.75 (0.72, 0.79)**			**0.78 (0.75, 0.81)**		

Supplementary Figure A: Assessing calibration of the apparent model in original data of the prediction of respiratory hospital admissions within 2 years

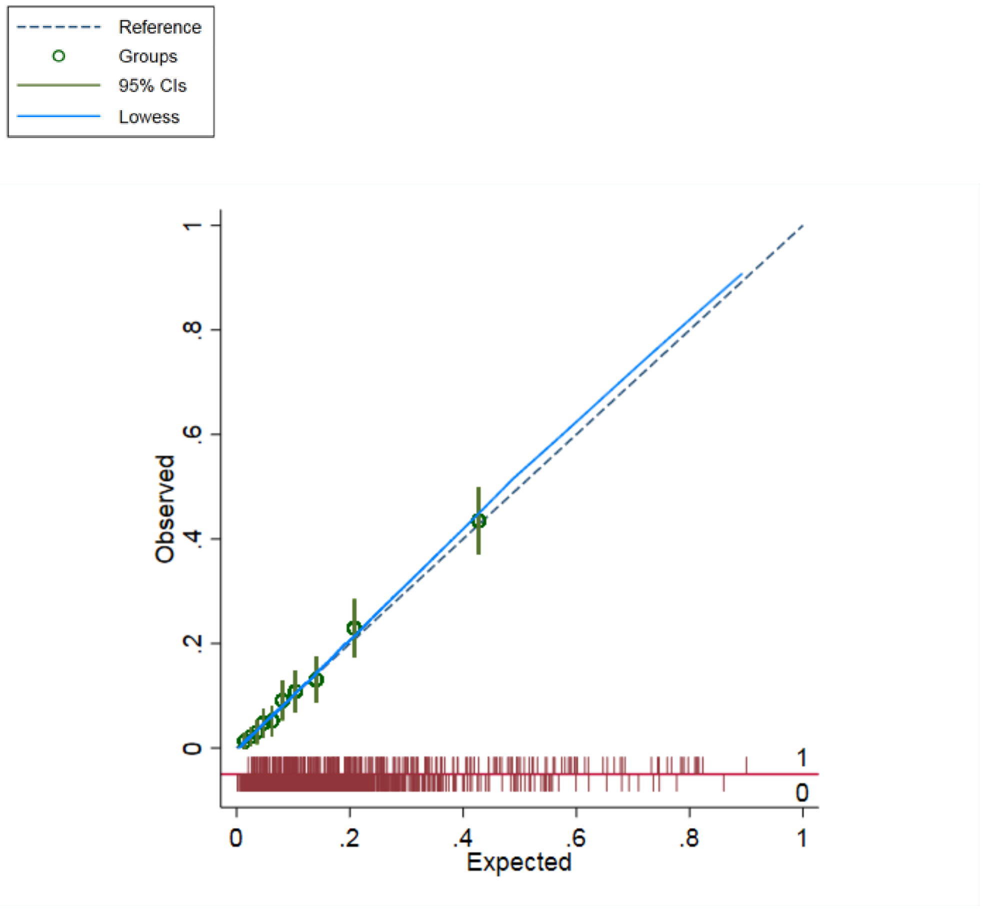

Red lines indicate individual respiratory admission events

Supplementary Figure B: Assessing calibration in the CPRD validation cohort of the prediction of respiratory hospital admissions within 2 years

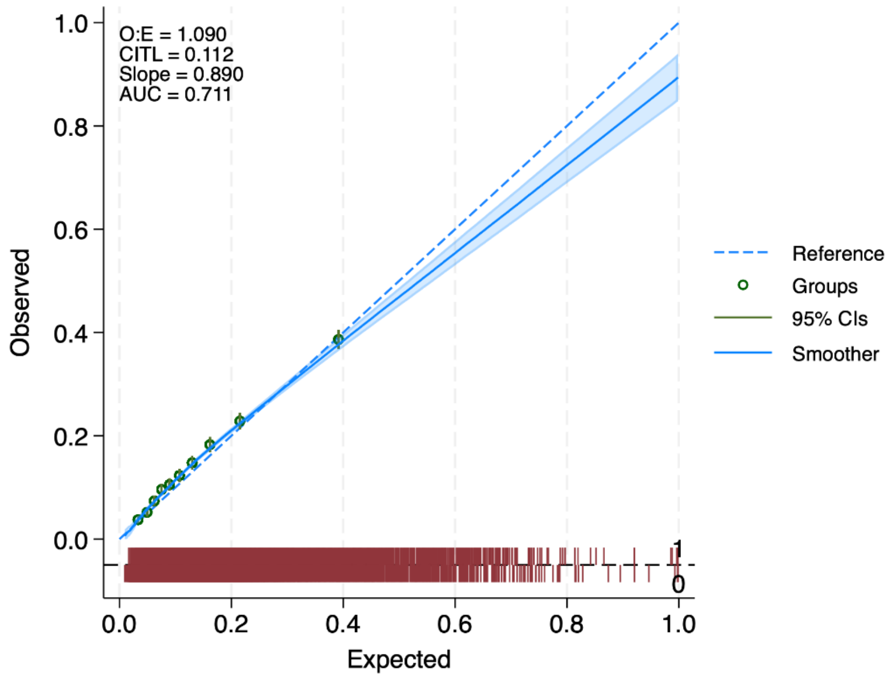

Red lines indicate individual respiratory admission events

Supplementary Figure C: Comparing performance of BLISS and Bertens' scores over one, two and three-year follow-up in the ECLIPSE cohort

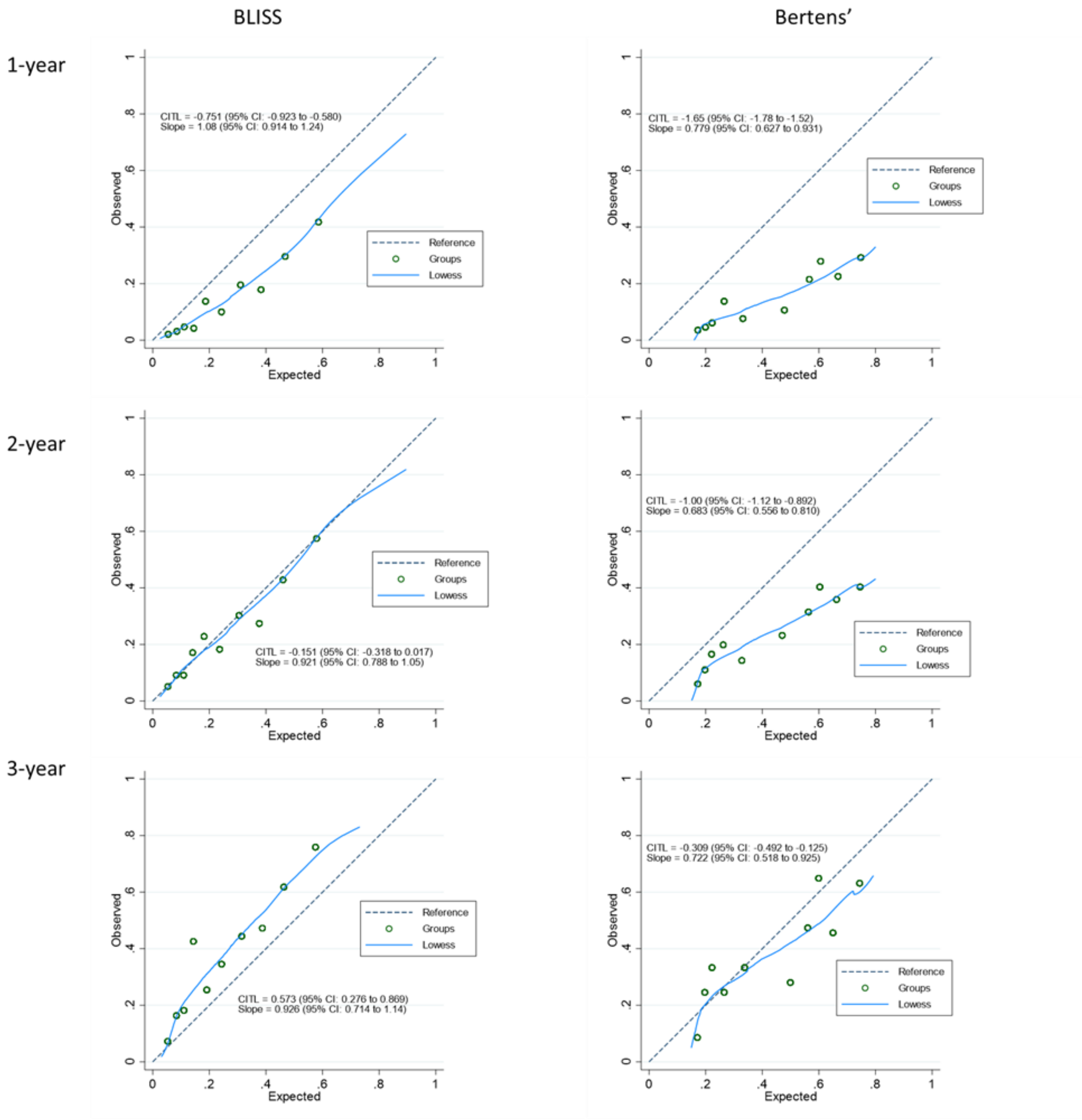

Supplementary Figure D: Net benefit of management using the BLISS score in the BLISS development dataset to stratify risk, compared with individual components of the score, treating all as high risk or treating none

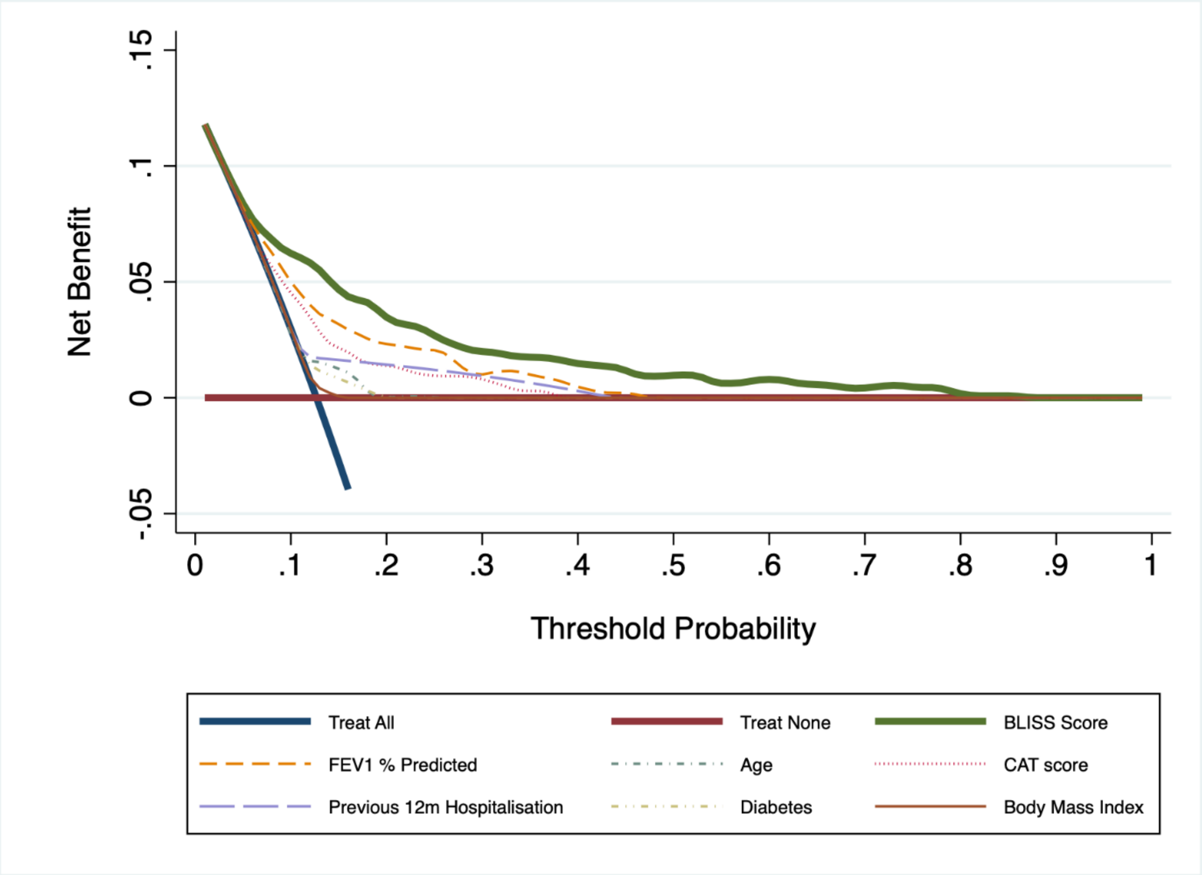

Supplementary Figure E: Net benefit of management using the BLISS score in the CPRD external validation dataset to stratify risk, compared with individual components of the score, treating all as high risk or treating none

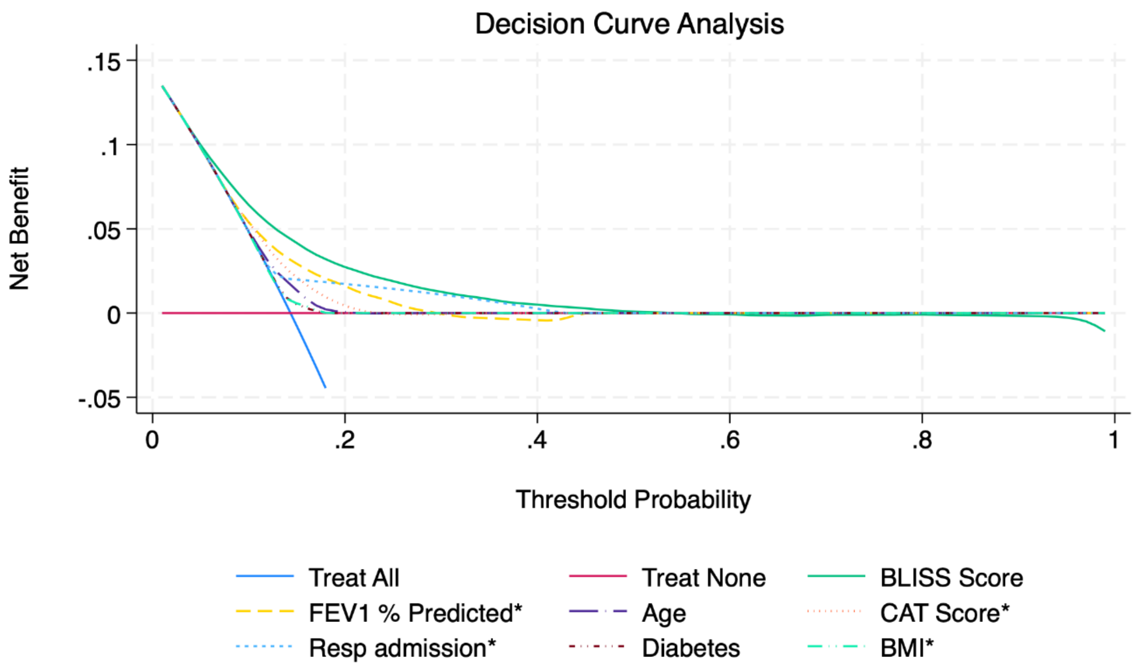

RESULTS OF SENSITIVITY ANALYSES DURING THE DEVELOPMENT OF THE BLISS SCORE

When including the additional 4 variables into the candidate variable pool, 8 predictors were retained: age, sex, CAT score, previous respiratory admission, BMI, self-report of diabetes and two measures of FEV_1 (FEV_1/height^2 and FEV_1Q) - apparent c-statistic 0.76 (0.73 to 0.80). This compares with the apparent performance of the primary model prior to optimism adjustment (c=0.76 (0.72,0.79)) (suppl Table 2) and 0.77 (0.73, 0.80) for the full model with all predictors.

Including only prevalent cases resulted in an apparent c-statistic of 0.75 (95%CI: 0.72 to 0.79), with smoking status included in addition. Including the full follow-up data resulted in an apparent c-statistic of 0.78 (95%CI: 0.75 to 0.81), with smoking status and heart failure additionally retained.
